# *Shigella* induces epigenetic reprogramming of zebrafish neutrophils

**DOI:** 10.1126/sciadv.adf9706

**Published:** 2023-09-06

**Authors:** Margarida C. Gomes, Dominik Brokatzky, Magdalena K. Bielecka, Fiona C. Wardle, Serge Mostowy

**Affiliations:** ^1^ Department of Infection Biology, London School of Hygiene and Tropical Medicine, London, UK.; ^2^ Randall Centre for Cell and Molecular Biophysics, New Hunt's House, Guy's Campus, King's College London, UK.

## Abstract

Trained immunity is a long-term memory of innate immune cells, generating an improved response upon reinfection. *Shigella* is an important human pathogen and inflammatory paradigm for which there is no effective vaccine. Using zebrafish larvae, we demonstrate that after *Shigella* training, neutrophils are more efficient at bacterial clearance. We observe that *Shigella*-induced protection is nonspecific and has differences with training by BCG and β-glucan. Analysis of histone ChIP-seq on trained neutrophils revealed that *Shigella* training deposits the active H3K4me3 mark on promoter regions of 1612 genes, dramatically changing the epigenetic landscape of neutrophils toward enhanced microbial recognition and mitochondrial ROS production. Last, we demonstrate that mitochondrial ROS plays a key role in enhanced antimicrobial activity of trained neutrophils. It is envisioned that signals and mechanisms we discover here can be used in other vertebrates, including humans, to suggest new therapeutic strategies involving neutrophils to control bacterial infection.

## Teaser

*Shigella* trains the zebrafish innate immune system to produce neutrophils with enhanced antimicrobial function via mtROS.

## INTRODUCTION

Trained immunity is an immunological memory characterized by remodeling of the epigenetic landscape and metabolism of myeloid cells, conferring enhanced responses upon a new infection challenge (*1*). The most commonly used training stimuli are *Mycobacterium bovis* Bacille Calmette-Guérin (BCG) and the fungal wall component β-glucan (*1*); however, reports have described a large breadth of triggers that include metabolites, inflammatory cytokines, and danger signals (*2*–*7*). Although work in vitro, ex vivo, in mice and in humans has mostly been centered around monocytes/macrophages (*1*, *8*), growing evidence shows that mechanisms of trained immunity can be observed in other immune and non-immune cells (such as endothelial and epithelial cells) (*9*–*11*). Recent reports have started to investigate training mechanisms by BCG and β-glucan in neutrophils (*12*, *13*); however, the impact of trained neutrophils at a whole animal level is mostly unknown.

The zebrafish embryo has been widely used as a model for developmental biology, cell biology, and infectious diseases (*14*). Zebrafish are genetically tractable, share >80% of human genes associated with diseases, and strictly depend on innate immune responses until 4 weeks postfertilization when the adaptive immune system starts to develop (*15*, *16*). Seminal studies have used zebrafish as a model to study hematopoietic stem and progenitor cell (HSPC) development and hematopoiesis (*17*–*19*), and the repertoire of innate immune cells in zebrafish is now well characterized (*20*, *21*). Considering this, the zebrafish model presents itself as an attractive model to study trained immunity. The first evidence that training mechanisms may also be conserved in fish comes from decades of use of β-glucans in aquaculture, where their use improved the resistance of teleost fish to infections (*22*). Further work with carp macrophages (*23*), vaccine development (*24*–*26*), and pathogenic infections (*19*, *27*, *28*) support the use of fish as a model to dissect fundamental determinants of trained immunity.

*Shigella* is an important human pathogen, among the top 12 priority pathogens requiring urgent action by the World Health Organization (*29*), and for which there are no effective vaccines (*30*). *Shigella* infection is well known to induce inflammation, by its lipopolysaccharide (LPS) and type III secretion system (T3SS) (*31*, *32*). We previously demonstrated that hallmarks of *Shigella* infection, including inflammation and macrophage cell death, can be observed using a zebrafish infection model (*33*, *34*). Work has also shown that a nonlethal *Shigella* infection triggers immune responses that lead to emergency granulopoiesis and protects zebrafish larvae from secondary infection (*28*, *35*).

Here, we introduce the zebrafish larvae infection model as a powerful system to study innate immune training. We show that *Shigella* training of zebrafish larvae generates protective neutrophils, and the training mechanism differs from that of BCG or β-glucan training. Genome-wide sequencing of epigenetic modifications in trimethylation at histone 3 lysine 4 (H3K4me3) in *Shigella*-trained neutrophils and downstream functional characterization shows that their epigenetic landscape is modified to increase mitochondrial reactive oxygen species (mtROS) production to better fight secondary infectious challenges.

## RESULTS

### Zebrafish training with a nonlethal dose of *Shigella* induces generation of protective neutrophils

**Fig. 1.**
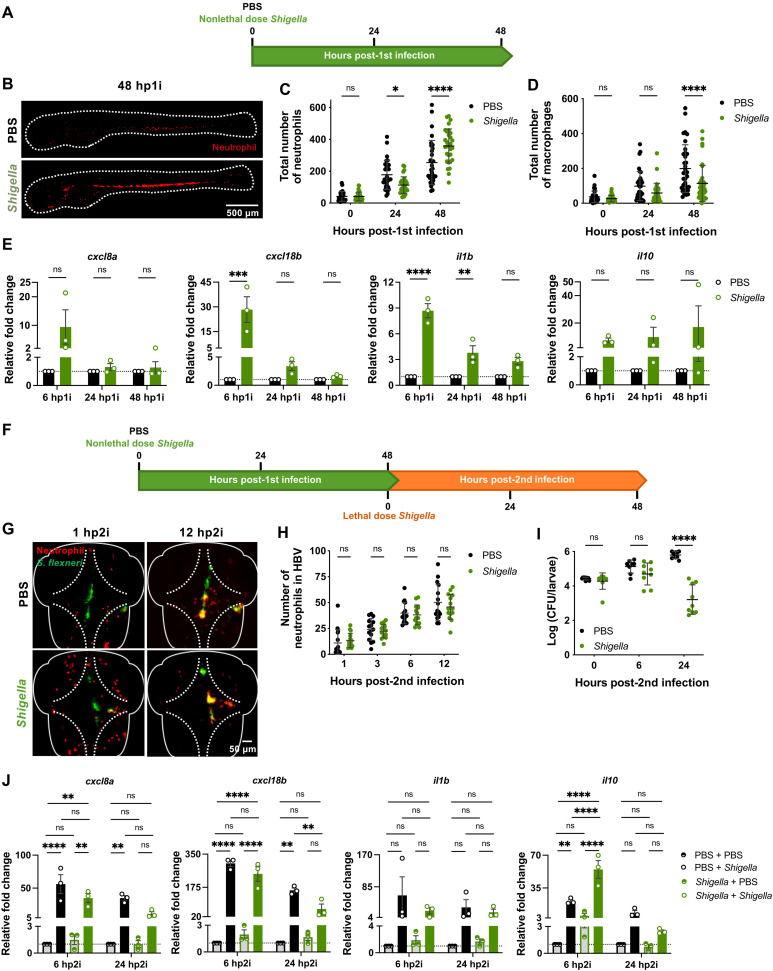
Zebrafish exposure to nonlethal *Shigella* dose induces generation of more resilient neutrophils. (**A**) Training of 2 dpf zebrafish larvae. (**B**) Representative images of Tg(*lyz*::dsRed) larvae at 48 hp1i. Scale bar, 500 μm. (**C**) Quantification of neutrophils [Tg(*lyz*::dsRed)] in naïve and *Shigella*-trained (2 × 10^3^ ± 1.4 × 10^3^ CFUs) larvae. *N* = 3 with 10 larvae per time point (mean ± SD). (**D**) Quantification of macrophages [Tg(*mpeg*::mCherry)] in naïve and *Shigella*-injected (1.8 × 10^3^ ± 8.6 × 10^2^ CFUs) larvae. *N* = 3 with 11 larvae per time point (mean ± SD). (**E**) Expression of *cxcl8a*, *cxcl18b*, *il1b*, and *il10* in *Shigella*-trained larvae (1.3 × 10^3^ ± 5.5 × 10^2^ CFUs) as compared to naïve larvae. *N* = 3 with >5 larvae per time point (mean ± SEM). (**F**) Reinfection of trained larvae with lethal dose injection of *Shigella* at 4 dpf. (**G**) Representative images of Tg(*lyz*::dsRed) naïve and *Shigella*-trained larvae reinfected with *Shigella flexneri* M90T. Scale bar, 50 μm. (**H**) Quantification of recruited neutrophils [Tg(*lyz*::dsRed)] to the HBV in naïve and *Shigella*-trained larvae following reinfection with *Shigella* (PBS, 2.6 × 10^4^ ± 9 × 10^3^ CFUs; *Shigella*, 2.7 × 10^4^ ± 1 × 10^4^ CFUs). *N* = 3 with 5 larvae per time point (mean ± SD). (**I**) Bacterial counts from naïve and *Shigella*-trained larvae reinfected with *Shigella* (PBS, 2.7 × 10^4^ ± 4.5 × 10^3^ CFUs; *Shigella*, 2.5 × 10^4^ ± 1 × 10^4^ CFUs). *N* = 3 with 3 larvae per time point (mean ± SD). (**J**) Expression of *cxcl8a*, *cxcl18b*, *il1b*, and *il10* following reinfection with *Shigella* in naïve (2.5 × 10^4^ ± 1 × 10^4^ CFUs) and *Shigella*-trained (2.7 × 10^4^ ± 4.5 × 10^3^ CFUs) as compared to PBS-injected controls. *N* = 3 with >5 larvae per time point (mean ± SEM). *P < 0.05, **P < 0.01, ***P < 0.001, ****P < 0.0001, two-way analysis of variance (ANOVA) with Sidak’s multiple comparisons test (C, D, H, I, and J). ns, not significant.

We previously demonstrated that a nonlethal dose of *Shigella flexneri* injected in the hindbrain ventricle (HBV) of zebrafish larvae at 2 days postfertilization (dpf) is rapidly cleared and induces HSPC proliferation and differentiation as well as emergency granulopoiesis in the aorta-gonad-mesonephros (AGM) region (*28*). To better understand the immune responses during a nonlethal *Shigella* infection, we quantified the total number of neutrophils and macrophages in larvae injected with ~2 × 10^3^ colony-forming units (CFU) (Fig. 1A). Neutrophil numbers significantly decrease at 24 hours post–first infection (hp1i), and by 48 hp1i, the pool of neutrophils is replenished in excess, consistent with observed granulopoiesis in the AGM (Fig. 1, B and C). Although the macrophage population is also significantly reduced by 24 hp1i, it does not recover by 48 hp1i (Fig. 1D), suggesting that macrophage hematopoiesis is impaired to favor granulopoiesis, consistent with previous reports (*3*, *6*, *36*, *37*).

Analysis of cytokine expression showed a significant increase in proinflammatory cytokines CXCL8a, CXCL18b, interleukin-1β (IL-1β), IL-6, and tumor necrosis factor–α (TNF-α) between 6 and 24 hp1i (Fig. 1E and fig. S1A), which correlates with neutrophil recruitment and bacterial clearance (fig. S1B). At 48 hp1i, the proinflammatory response induced by *Shigella* training returns to basal levels, but expression of the anti-inflammatory cytokine IL-10 remains elevated. These results are consistent with the requirement of inflammation to induce proliferation of HSPCs (*18*, *19*, *28*) and show that trained larvae (i.e., those injected with a nonlethal dose of *Shigella*) are at immunological homeostasis before reinfection.

Upon a second lethal *Shigella* challenge (Fig. 1F), trained larvae show significantly higher survival rates by 48 hours post–second infection (hp2i) as compared to naïve larvae [i.e., those injected with phosphate-buffered saline (PBS)]. Quantification of neutrophil recruitment to the HBV when infected with a lethal dose (>2 × 10^4^ CFU) showed that recruitment in *Shigella*-trained larvae is not significantly different from naïve larvae (Fig. 1, G and H). At 6 hp2i, bacterial burden in *Shigella*-trained larvae starts to reduce, as compared to naïve larvae (Fig. 1I), suggesting that trained neutrophils are better at clearing bacteria. Similarities in neutrophil recruitment in both conditions during infection are consistent with expression of proinflammatory cytokines CXCL8a and CXCL18b, which are expressed to the same extent in naïve and trained larvae at 6 hp2i (Fig. 1J). In trained infected larvae, the anti-inflammatory cytokine IL-10 expression is significantly different compared to that in naïve larvae (both uninfected and infected), suggesting that trained larvae strongly induce anti-inflammatory responses. At 24 hp2i, all analyzed cytokines are less expressed in trained infected larvae compared to naïve infected larvae (Fig. 1J and fig. S1C). Together, these observations indicate that *Shigella*-trained larvae are protected against a secondary infectious challenge because of tighter control of immune responses and their neutrophils have increased killing capacity, which are important characteristics of innate immune training.

### *Shigella* induces nonspecific and lasting training mechanisms in zebrafish larvae

**Fig. 2.**
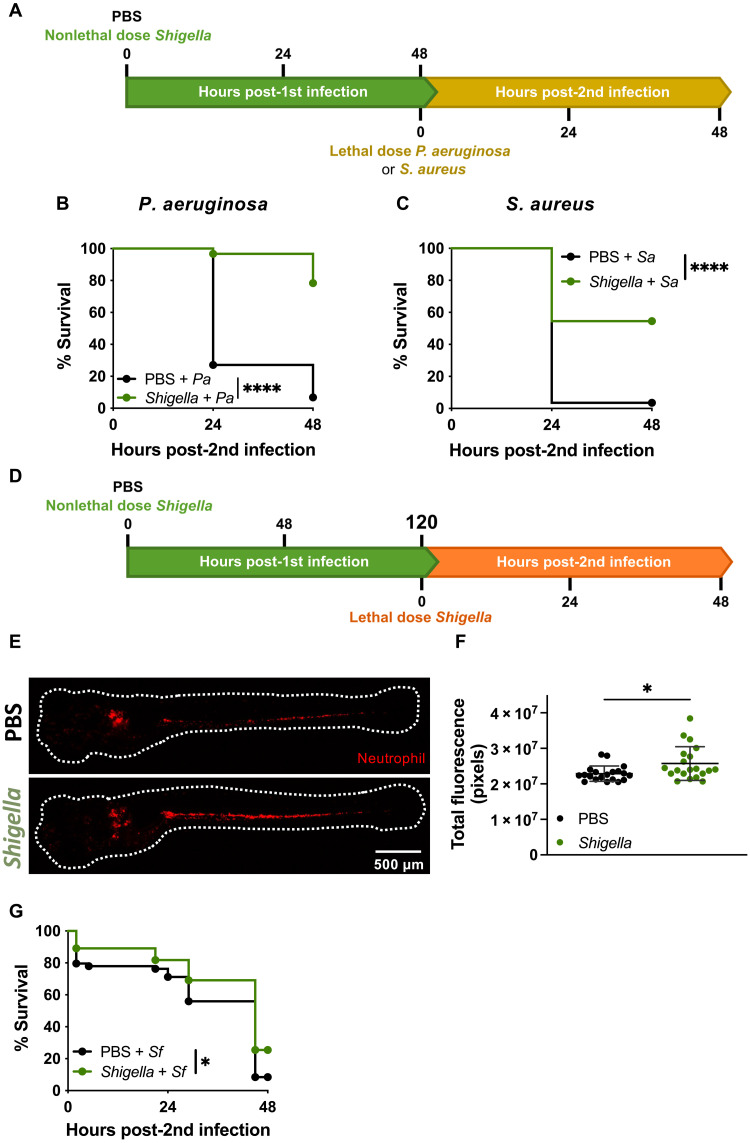
*Shigella* induced protection is nonspecific. (**A**) Reinfection of trained larvae with lethal dose injection of *P. aeruginosa* (*Pa*) or *S. aureus* (*Sa*) at 4 dpf. (**B**) Survival curves from naïve and *Shigella*-trained (2.2 × 10^3^ ± 3.8 × 10^2^ CFUs) larvae infected with *P. aeruginosa* (PBS, 2.6 × 10^3^ ± 7.4 × 10^2^ CFUs; *Shigella*, 2.3 × 10^3^ ± 5.5 × 10^2^ CFUs). *N* = 3 with 20 larvae per experiment. (**C**) Survival curves from naïve and *Shigella*-trained (1.4 × 10^3^ ± 8.6 × 10^2^ CFUs) larvae infected with *S. aureus* (PBS, 3.4 × 10^4^ ± 1.1 × 10^4^ CFUs; *Shigella*, 3.4 × 10^4^ ± 6 × 10^3^ CFUs). *N* = 3 with >20 larvae per experiment. (**D**) Reinfection of trained larvae with lethal dose injection of *Shigella* at 7 dpf (or 5 dp1i). (**E**) Representative images of naïve and *Shigella*-trained Tg(*lyz*::dsRed) larvae with at 5 dp1i. Scale bar, 500 μm. (**F**) Quantification of total fluorescence in Tg(*lyz*::dsRed) naïve and *Shigella*-trained (1.7 × 10^3^ ± 5.7 × 10^2^ CFUs) larvae at 5 dp1i. *N* = 3 with 10 larvae per time point (mean ± SD). (**G**) Survival curves from naïve and *Shigella*-trained larvae infected with *Shigella* at 5 dp1i. *N* = 3 with >16 larvae per experiment. *P < 0.05, ****P < 0.0001, log-rank (Mantel-Cox) test (B, C, and G) and unpaired Student’s *t* test (F).

Work on trained innate immunity using BCG has demonstrated that induced protection is not specific to *Mycobacterium tuberculosis* and helps in controlling fungal infection by *Candida albicans* (*38*). In zebrafish, β-glucan and *Salmonella enterica* serovar Typhimurium have been shown to protect against Gram-negative and Gram-positive pathogens (*27*). Here, we tested our *Shigella*-trained larvae against lethal infections of *Pseudomonas aeruginosa* (a Gram-negative pathogen, 2 × 10^3^ CFU) and *Staphylococcus aureus* (a Gram-positive pathogen, 2 × 10^4^ CFU) (Fig. 2A). In both cases, the survival of *Shigella*-trained larvae is significantly higher as compared to the survival of naïve larvae (Fig. 2, B and C), and bacterial burden is controlled from 6 hp2i (fig. S2, A and B). These results demonstrate that *Shigella*-induced protection of zebrafish is nonspecific.

To test the longevity of protection induced by *Shigella* training, we increased the resting interval between training and reinfection from 2 to 5 days (Fig. 2D). At this time point (7 dpf), we observed that neutrophil numbers remain higher in trained larvae as compared to naïve larvae (Fig. 2, E and F), but the differences are smaller than at 4 dpf (Fig. 1, A to C). Upon reinfection with a lethal dose of *Shigella* (2.5 × 10^4^ CFU), survival of trained larvae is significantly higher compared to naïve larvae (Fig. 2G). However, differences in bacterial clearance are less detectable at 24 hp2i between groups (fig. S2C).

### BCG and β-glucan induce protection in zebrafish larvae

**Fig. 3.**
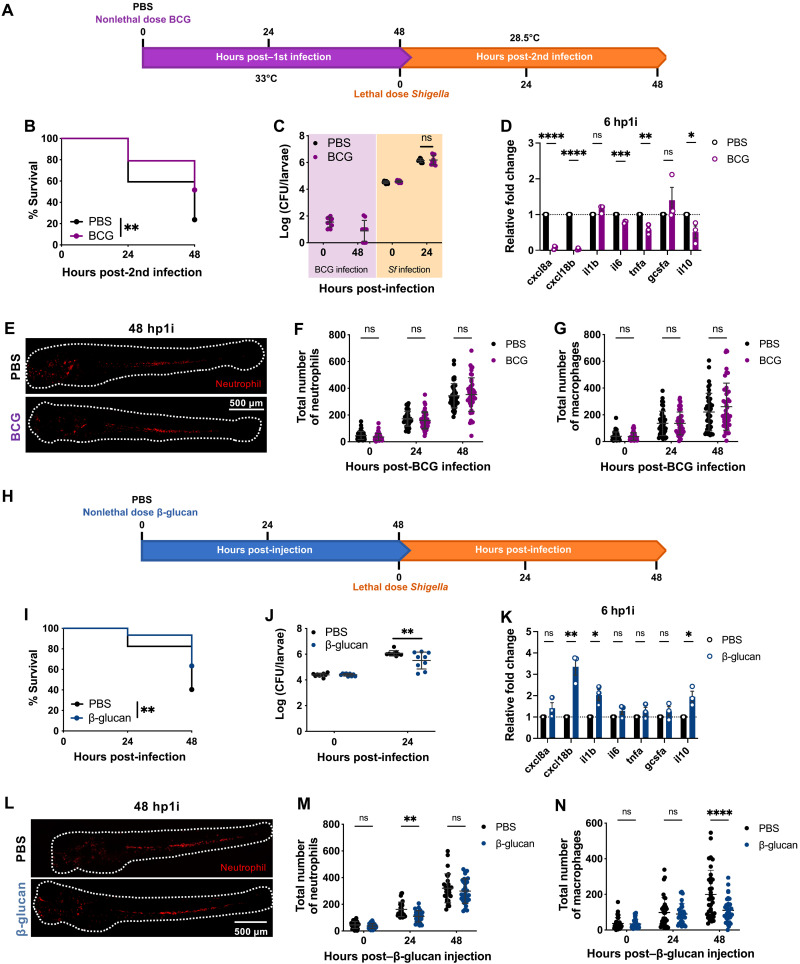
BCG and β-glucan induce protection in zebrafish embryos. (**A**) Reinfection of BCG-trained larvae with *Shigella* at 4 dpf. (**B**) Survival curves (*N* = 3 with >16 larvae per experiment) and (**C**) bacterial counts [*N* = 3 with 3 larvae per time point (mean ± SD)] from naïve and BCG-trained larvae infected with *Shigella*. (**D**) Expression of *cxcl8a*, *cxcl18b*, *il1b*, *il6*, *tnfa*, *gcsfa*, and *il10* in BCG-trained larvae (4.7 × 10^1^ ± 3.2 × 10^1^ CFUs) as compared to naïve larvae. *N* = 3 with 10 larvae per time point (mean ± SEM). (**E**) Representative images of naïve and BCG-trained Tg(*lyz*::dsRed) larvae at 48 hp1i. Scale bar, 500 μm. (**F**) Quantification of neutrophils [Tg(*lyz*::dsRed)] and (**G**) macrophages [Tg(*mpeg*::mCherry)] in naïve and BCG-trained larvae [(F) 5.6 × 10^1^ ± 4.3 × 10^1^ CFUs, (G) 6.3 × 10^1^ ± 3.7 × 10^1^ CFUs]. *N* = 3 with >12 larvae per experiment (mean ± SD). (**H**) Reinfection of β-glucan–trained larvae with *Shigella* at 4 dpf. (**I**) Survival curves (*N* = 3 with >18 larvae per experiment) and (**J**) bacterial counts [*N* = 3 with 3 larvae per time point (mean ± SD)] from naïve and β-glucan–trained (20 ng) larvae infected with *Shigella*. (**K**) Expression of *cxcl8a*, *cxcl18b*, *il1b*, *il6*, *tnfa*, *gcsfa*, and *il10* in β-glucan–trained larvae (20 ng) as compared to naïve larvae. *N* = 3 with 10 larvae per time point (mean ± SEM). (**L**) Representative images of naïve and β-glucan–trained Tg(*lyz*::dsRed) larvae at 48 hp1i. Scale bar, 500 μm. (**M**) Quantification of neutrophils [Tg(*lyz*::dsRed)] and (**N**) macrophages [Tg(*mpeg*::mCherry)] in naïve and β-glucan–trained larvae (20 ng). *N* = 3 with >8 larvae per experiment (mean ± SD). *P < 0.05, **P < 0.01, ***P < 0.001, ****P < 0.0001, log-rank (Mantel-Cox) test (B and I), one-way ANOVA with Tukey’s multiple comparisons test (C and J), unpaired Student’s *t* test (D and K), and two-way ANOVA with Sidak’s multiple comparisons test (F, G, M, and N).

BCG and β-glucan have been widely studied as inducers of trained immunity (*38*, *39*). To better understand the mechanisms by which *Shigella* can induce trained immunity, we developed zebrafish reinfection models with BCG and β-glucan for comparison. In the BCG reinfection model (Fig. 3A), a low dose of BCG (50 CFU) was injected in 2 dpf larvae that were then incubated at 33°C for 48 hours to stimulate BCG replication (*40*). Although BCG were not completely cleared from larvae (Fig. 3C), reinfection with a lethal dose of *Shigella* was done at 48 hp1i, and survival was followed for another 48 hours at 28.5°C. In this case, BCG-trained larvae had significantly greater survival than naïve larvae (Fig. 3B), although they presented a similar burden of *Shigella* at 24 hp2i (Fig. 3C). The difference between BCG and *Shigella* training may be associated with proinflammatory responses that are induced during training. Whereas *Shigella* strongly induces proinflammatory responses by 6 hp1i (Fig. 1E), BCG infection does not (Fig. 3D). Unexpectedly, the expressions of neutrophil chemokines CXCL8a and CXCL18b were repressed at 6 hp1i, which may explain the lack of neutrophil recruitment to the HBV by 24 hp1i (fig. S3A). Macrophages, in contrast, were recruited to the injection site and remained in the HBV until 24 hp1i (fig. S3B). Overall, no differences in neutrophil and macrophage numbers were observed during BCG infection (Fig. 3, E to G).

To establish a β-glucan (local) reinfection model (Fig. 3H), 20 ng of β-glucan was injected in the HBV of larvae and infection with *Shigella* occurred 48 hours after. Larvae trained with β-glucan had increased survival (Fig. 3I) and reduced bacterial burden by 24 hp2i (Fig. 3J). Analysis of cytokine expression at 6 hp1i demonstrated that *cxcl18b*, *il1b*, and *il10* are significantly more expressed in β-glucan–trained larvae compared to naïve larvae (Fig. 3K), suggesting that β-glucan is recognized by the immune system. Similar to *Shigella* training, β-glucan induced changes in the neutrophil and macrophage populations. At 24 hp1i, the neutrophil numbers were significantly reduced, and by 48 hp1i, the population was restored to similar numbers as in naïve larvae (Fig. 3, L to M). The granulopoiesis observed was associated to expression of *gcsfa* expression at 24 hp1i (fig. S3F), but there was no significant increase in the number of neutrophils in the AGM as compared to naïve larvae (fig. S3G). Consequently, and similar to *Shigella* training, macrophage differentiation was blunted by 48 hp1i (Fig. 3N).

Considering differences across zebrafish models of trained innate immunity (Table 1), we conclude that the training mechanism induced by *Shigella* is different from that of BCG and β-glucan. However, the different training mechanisms share important protective phenotypes, such as the reduction of pathogen burden (independently of the bacterial species) and improvement of host survival.

**Table 1.** Comparison of training phenotypes between *Shigella*-, BCG-, and β-glucan–trained zebrafish larvae.

		*Shigella*	BCG	β-glucan
**Training**	Bacterial clearance	Yes	No	–
	Neutropenia (24 hp1i)	Yes	No	Yes
	Emergency granulopoiesis (48 hp1i)	Yes	No	No
	Changes in macrophage population	Yes	No	Yes
	Induction proinflammatory responses	Yes	No	Yes
**Infection challenge**	Survival protection	Yes	Yes	Yes
	Infection control	Yes	No	Yes
	Nonspecific	Yes	Yes	Yes

### Contribution of host factors, LPS, and *Shigella* effectors to training mechanisms

*Gcsf* signaling is crucial to induce emergency granulopoiesis in *Shigella*-trained larvae (*28*); however, it is not the only requirement to induce training mechanisms. To better understand triggers for *Shigella*-induced training in zebrafish larvae, we tested different factors important in *Shigella* infection of host cells (Fig. 3).

**Fig. 4.**
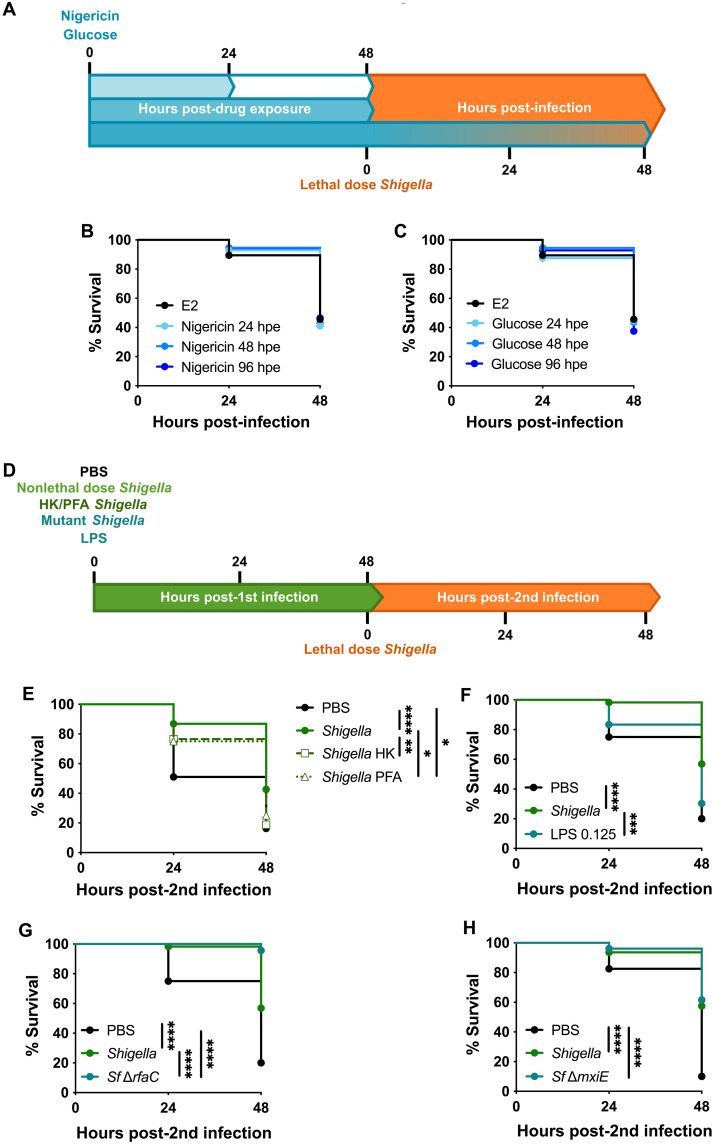
*Shigella* stimulates inflammatory pathways to induce training. (**A**) Exposure of zebrafish larvae to nigericin and glucose followed by *Shigella* infection. (**B**) Survival curves from E2- and nigericin (0.1 μM)–treated larvae infected with *Shigella* (PBS, 2.7 × 10^4^ ± 8.5 × 10^3^ CFUs; nigericin, 2.6 × 10^4^ ± 4.1 × 10^3^ CFUs). *N* = 3 with >14 larvae per experiment. hpe, hours post-drug exposure (**C**) Survival curves from E2- and glucose (1%)–treated larvae infected with *Shigella* (PBS, 2.7 × 10^4^ ± 8.5 × 10^3^ CFUs; glucose, 2.7 × 10^4^ ± 1 × 10^4^ CFUs). *N* = 3 with >14 larvae per experiment. (**D**) Reinfection of larvae trained with nonlethal dose of live or killed *Shigella*, *Shigella* mutants, or LPS followed by infection with *Shigella* at 4 dpf. (**E**) Survival curves from naïve, live-*Shigella*–trained (1.3 × 10^3^ ± 6.6 × 10^2^ CFUs), *Shigella* heat-killed (HK)–trained, and *Shigella* PFA–trained larvae infected with *Shigella* (PBS, 2.7 × 10^4^ ± 6.1 × 10^3^ CFUs; *Shigella*, 2.7 × 10^4^ ± 1 × 10^4^ CFUs; *Shigella* HK, 3.2 × 10^4^ ± 3.3 × 10^4^ CFUs; *Shigella* PFA, 3.2 × 10^4^ ± 1.7 × 10^4^ CFUs). *N* = 3 with >11 larvae per experiment. (**F**) Survival curves from naïve *Shigella*-trained (1.6 × 10^3^ ± 6.5 × 10^2^ CFUs) and LPS-primed (0.125 ng) larvae infected with *Shigella* (PBS, 1.9 × 10^4^ ± 6.3 × 10^3^ CFUs; *Shigella*, 2.8 × 10^4^ ± 4.7 × 10^3^ CFUs; LPS, 1.9 × 10^4^ ± 9.1 × 10^3^ CFUs). *N* = 3 with >18 larvae per experiment. (**G**) Survival curves from naïve, *Shigella*-trained (1.6 × 10^3^ ± 6.5 × 10^2^ CFUs), and *Shigella* Δ*rfaC*–trained (8.5 × 10^2^ ± 7.5 × 10^2^ CFUs) larvae infected with *Shigella* (PBS, 1.9 × 10^4^ ± 6.3 × 10^3^ CFUs; *Shigella*, 2.8 × 10^4^ ± 4.7 × 10^3^ CFUs; Δ*rfaC*, 2.4 × 10^4^ ± 8.4 × 10^3^ CFUs). *N* = 3 with >10 larvae per experiment. (**H**) Survival curves from naïve, *Shigella*-trained (7.9 × 10^2^ ± 5.5 × 10^2^ CFUs) and *Shigella* Δ*mxiE*–trained (8.8 × 10^2^ ± 5.8 × 10^2^ CFUs) larvae infected with *Shigella* (PBS, 1.9 × 10^4^ ± 7.5 × 10^3^ CFUs; *Shigella*, 2.5 × 10^4^ ± 4.6 × 10^3^ CFUs; Δ*mxiE*, 2.2 × 10^4^ ± 7.6 × 10^3^ CFUs). *N* = 3 with >10 larvae per experiment. *P < 0.05, **P < 0.01, ***P < 0.001, ****P < 0.0001, log-rank (Mantel-Cox) test (B, C, E, F, G, and H).

As previously described (*18*), inflammasome activation plays an important role in HSPCs production, and *Shigella* has been shown to be a potent activator of inflammasome pathways (*31*, *32*). Therefore, we tested whether NOD-, LRR- and pyrin domain–containing protein 3 (NLRP3) inflammasome activation by nigericin and glucose in HSPCs is sufficient to induce training. Zebrafish larvae were bathed in either 0.1 μM nigericin or 1% glucose for at least 24 hours from 2 dpf, and at 4 dpf, larvae were injected with a lethal dose of *Shigella* (Fig. 4A). The survival assay showed that independently of exposure time to either nigericin or glucose, no differences in survival (Fig. 4, B and C) or bacterial burden (fig. S4, A and B) are observed. These results suggest that inflammasome and caspase-1 activation is not sufficient to induce training.

Recently, studies with BCG and *Salmonella* have shown that inactivated or heat-killed bacteria can induce trained immunity mechanisms; however, the protection observed upon second challenge is weaker (*27*, *40*, *41*). In the case of *Shigella* (Fig. 4D), both heat-killed (HK) and paraformaldehyde (PFA)–killed *Shigella* failed to protect larvae survival upon a secondary lethal dose of *Shigella* (Fig. 4E and fig. S4C), strongly suggesting that *Shigella* needs to be alive to induce training.

To test the role of the LPS in training, LPS was used to prime larvae followed by secondary challenge with a lethal dose of *Shigella* (Fig. 4F and fig. S4, D to F). Larvae survival upon a lethal *Shigella* injection was not significantly affected by priming with LPS, highlighting that in our model LPS alone does not induce protection mechanisms. However, complete exposure of lipid A in a *Shigella* ∆*rfaC* mutant [which lacks the O-antigen, outer and inner core of LPS (*42*)] induced 100% protection upon reinfection with *Shigella* (Fig. 4G) and highly efficient bacterial clearance (fig. S4G). Virulence assays in zebrafish larvae showed that 1 × 10^4^ CFU of *Shigella ∆rfaC* mutant can cause 100% lethality in 48 hours postinfection, whereas *Shigella* wild-type (WT) only kills 50% of the larvae (fig. S4H). The lethality was found to be caused by heighted proinflammatory responses and not due to bacterial burden (fig. S4, I and J). These results support work showing that stronger proinflammatory responses induced during training can result in better innate immune training (*40*).

Previous work has shown that the T3SS plays a role in training (*28*). Here, we tested the effect of T3SS effectors regulated by the master regulator MxiE (*43*). Larvae survival upon reinfection showed that MxiE-regulated effectors do not significantly affect how *Shigella* induces training (Fig. 4H and fig. S4K). We also tested a *Shigella* ∆*ospF* mutant, lacking the bacterial immunomodulin OspF (*44*, *45*), where similar results to *∆mxiE* mutant were obtained (fig. S4, L and M). These results are consistent with similar levels of virulence observed during infection of zebrafish larvae with *Shigella* ∆*mxiE* and ∆*ospF* mutants and *Shigella* WT (fig. S4, N to Q). Together, these data suggest that live *Shigella,* with a functional T3SS, is required to induce inflammatory responses that trigger training mechanisms.

### Exposure to *Shigella* induces epigenetic reprogramming of neutrophils

**Fig. 5.**
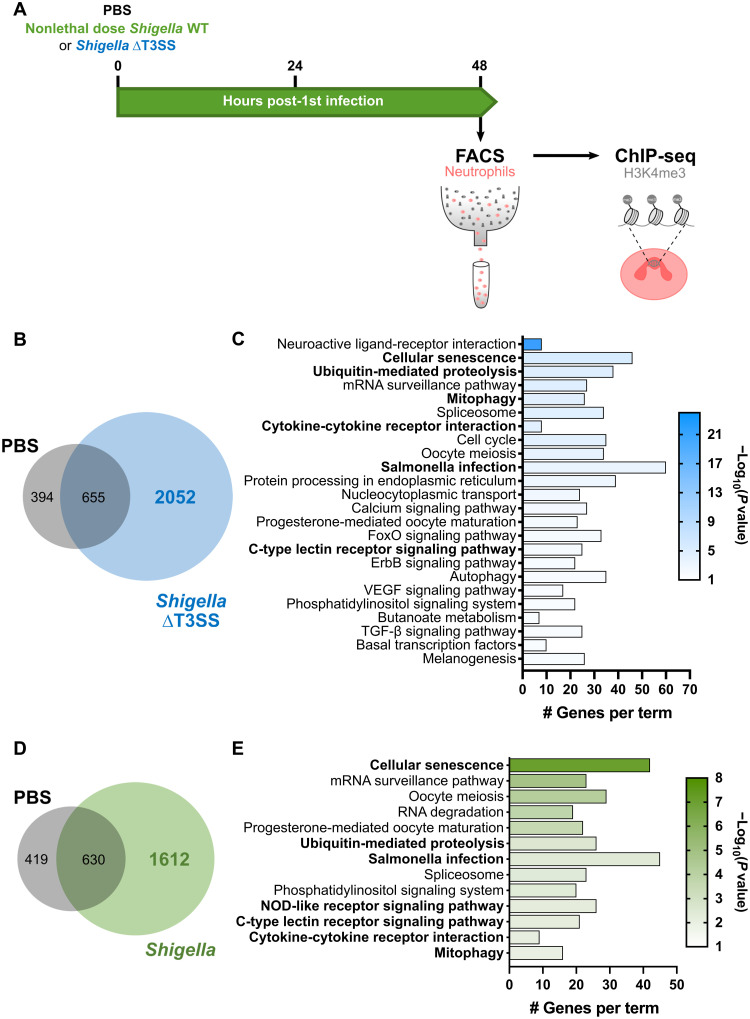
Exposure to *Shigella* induces epigenetic reprogramming of neutrophils. (**A**) Isolation of neutrophils by FACS from naïve and *Shigella*-trained larvae for ChIP-seq on H3K4me3 modification. (**B**) Venn diagram showing the number of common and unique genes marked by H3K4me3 peaks in their promoter regions [±3 kb from transcription start site (TSS)] of PBS and *Shigella* ∆T3SS–trained larvae. (**C**) Enriched Kyoto Encyclopedia of Genes and Genomes (KEGG) pathways (*P* value < 0.01) associated with the 2052 unique *Shigella* ∆T3SS training genes marked by an H3K4me3 peak in their promoter regions (±3 kb from TSS). FoxO, Forkhead box O; ErbB, erythroblastic oncogene B; VEGF, vascular endothelial growth factor; TGF-β, transforming growth factor–β. (**D**) Venn diagram showing the number of common and unique genes marked by H3K4me3 peaks in their promoter regions (±3 kb from TSS) of PBS and *Shigella* WT trained larvae. (**E**) Enriched KEGG pathways (*P* value < 0.01) associated with the 1612 unique *Shigella* WT training genes marked by an H3K4me3 peak in their promoter regions (±3 kb from TSS). NOD, nucleotide binding oligomerization domain.

Epigenetic reprogramming is a defining characteristic underlying the innate immune training of myeloid cells, and the enrichment of H3K4me3 on gene promoter regions is typically associated to active/poised gene transcription (*8*, *38*, *39*, *46*). Considering this, we investigated whether *Shigella*-trained neutrophils exhibit different H3K4me3 patterns that could explain their ability to better respond to reinfection. To test this, neutrophils were isolated by fluorescence-activated cell sorting (FACS) from naïve and trained [*Shigella* WT and *Shigella* ∆T3SS (i.e., *mxiD* mutant)] larvae at 48 hp1i (fig. S5A), and chromatin immunoprecipitation sequencing (ChIP-seq) was performed on the H3K4me3 mark (Fig. 5A). Genes were then annotated if they had a H3K4me3 mark in their promoter region [defined as ±3 kb from the transcription start site (TSS); data files S1 to S3]. Naïve neutrophils displayed H3K4me3 peaks at the promoter region of 1049 genes, which are associated with normal cell functioning (fig. S5, B and C), including surveying the quality of mRNA to increase the fidelity of RNA processing. Notably, neutrophil samples trained with *Shigella* WT or ΔT3SS showed an increase in the number of genes marked by H3K4me3 (2242 and 2707, respectively) as compared to naïve neutrophils, suggesting epigenetic remodeling of trained neutrophils (Fig. 5, B and D, and fig. S5, D and E).

Upon *Shigella* ∆T3SS training, pathway analysis of marked gene promoter regions indicates that some modulated responses are common to *Shigella* WT training; however, many other different signaling pathways are affected (Fig. 5C and data file S2), highlighting that training with *Shigella* WT leads to a more precise and efficient immune response. *Shigella* WT–induced H3K4me3 deposition at promoter regions was observed for genes involved in cellular senescence and in infection signaling pathways, specifically associated to infections by Gram-negative pathogens (Fig. 5E). The analysis suggests that trained neutrophils have metabolic and gene expression alterations linked to senescence and also activation of innate immune signaling via mitogen-activated protein kinase (MAPK) signaling pathway to express proinflammatory cytokines, chemokines, and antimicrobial peptides. In addition, although most promoter regions of proinflammatory cytokines are not marked by H3K4me3, deposition of this mark was found in promoter regions of cytokine receptor genes *il10rb*, *tgfbr1b*, *ifngr1l*, *cxcr3.2*, and *cxcr3.3* (data file S3). These results highlight the signaling pathways that may be activated and their importance for cell survival and immunomodulation.

### Reprogrammed neutrophils produce more mtROS for enhanced antimicrobial function

**Fig. 6.**
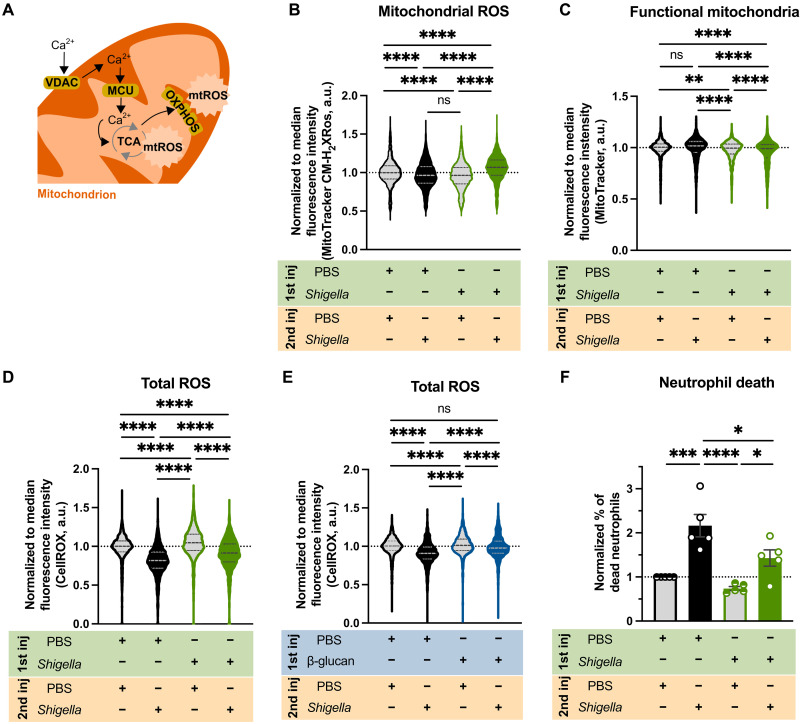
Trained neutrophils produce more mtROS. (**A**) Increased calcium transportation via VDAC and MCU increases mtROS. (**B**) Normalized fluorescence of MitoTracker Red CM-H_2_XRos in neutrophils from naïve and *Shigella*-trained (1.2 × 10^3^ ± 5.3 × 10^2^ CFUs) larvae [Tg(*mpx*::GFP)] unstimulated or infected with *Shigella* (PBS, 2.6 × 10^4^ ± 9.3 × 10^3^ CFUs; *Shigella*, 3 × 10^4^ ± 6.6 × 10^3^ CFUs) at 6 hp2i. *N* = 3 with >10 larvae per experiment (mean ± SD). (**C**) Normalized fluorescence of MitoTracker Deep Red in neutrophils from naïve and *Shigella*-trained (1.8 × 10^3^ ± 1 × 10^3^ CFUs) larvae [Tg(*mpx*::GFP)] unstimulated or infected with *Shigella* (PBS, 2.1 × 10^4^ ± 1.1 × 10^4^ CFUs; *Shigella*, 1.9 × 10^4^ ± 8.3 × 10^3^ CFUs) at 6 hp2i. *N* = 3 with 30 larvae per experiment (mean ± SD). (**D**) Normalized fluorescence of CellROX Deep Red in neutrophils from naïve and *Shigella*-trained (1.4 × 10^3^ ± 6.7 × 10^2^ CFUs) larvae [Tg(*mpx*::GFP)] unstimulated or infected with *Shigella* (PBS, 2.6 × 10^4^ ± 1 × 10^4^ CFUs; *Shigella*, 2.6 × 10^4^ ± 1 × 10^4^ CFUs) at 6 hp2i. *N* = 3 with >10 larvae per experiment (mean ± SD). (**E**) Normalized fluorescence of CellROX Deep Red in neutrophils from naïve and β-glucan–trained larvae [Tg(*mpx*::GFP)] unstimulated or infected with a lethal dose of *Shigella flexneri* M90T (PBS, 2 × 10^4^ ± 1.3 × 10^4^ CFUs; β-glucan, 2.7 × 10^4^ ± 9.8 × 10^3^ CFUs) at 6 hp2i. *N* = 3 with >20 larvae per experiment (mean ± SD). (**F**) Normalized percentage of dead neutrophils (LIVE/DEAD Fixable Violet Stain) from naïve and *Shigella*-trained larvae [Tg(*mpx*::GFP)] unstimulated or infected with *Shigella* at 6 hp2i. *N* = 5 with >10 larvae per experiment (mean ± SD). *P < 0.05, ***P < 0.001, ****P < 0.0001, one-way ANOVA with Tukey’s multiple comparisons test (B, C, D, and E). a.u., arbitrary units.

The ChIP-seq dataset strongly suggested increased mtROS production upon *Shigella* WT training (Fig. 5E). In agreement, we found that the promoter regions of the genes encoding mitochondrial calcium uniporter (MCU) and voltage-dependent anion channel (VDAC) transporters [mitochondrial permeability transition pore (mPTP)], as well as several genes involved in the tricarboxylic acid cycle and oxidative phosphorylation (Fig. 6A and data file S3), are marked by H3K4me3 in *Shigella* WT–trained but not in naïve larvae. To test whether mtROS production was increased in trained neutrophils, we used flow cytometry to quantify MitoTracker CM-H_2_XRos dye in naïve and *Shigella*-trained neutrophils in resting and restimulated state at 6 hp2i. Quantifications show that naïve neutrophils when infected with *Shigella* have reduced mtROS, compared to unstimulated neutrophils (Fig. 6B). Trained neutrophils also present lower levels of mtROS when unstimulated; however, after 6 hp2i, mtROS levels are significantly increased as compared to unstimulated naïve and trained neutrophils. To test for differences in mitochondrial mass between naïve and trained neutrophils, we measured functional mitochondria using MitoTracker staining (Fig. 6C). We found that naïve neutrophils exposed to infection (despite having more functional mitochondria) do not produce more mtROS to combat *Shigella*, as compared to unstimulated naïve neutrophils (Fig. 6, B and C, and fig. S6D). In contrast, trained neutrophils exposed to infection produce more mtROS without increasing the amount of functional mitochondria, as compared to unstimulated trained neutrophils.

Given the redox cross-talk between mitochondria and NADPH (reduced form of NADP^+^) oxidases (*47*), we investigated total ROS production in neutrophils using CellROX dye. Quantifications showed that naïve neutrophils when infected (6 hp2i) have significantly lower total ROS levels as compared to unstimulated state (Fig. 6D). *Shigella*-trained neutrophils in resting phase showed higher levels of ROS, compared to naïve unstimulated neutrophils. However, when trained neutrophils became stimulated, total ROS levels decreased, but not to the same extent as in naïve infected neutrophils. The effect of both mtROS and ROS on bacterial killing was tested by adding specific inhibitors of mPTP [cyclosporin A (CsA)] and NADPH oxidase [diphenyleneiodonium chloride (DPI)] to naïve and *Shigella*-trained larvae bath during the 6 hp2i. Recovered CFU showed that inhibitors had more impact on bacterial burden control in *Shigella*-trained larvae than in naïve larvae (fig. S6, B, C, F, and G), suggesting that both mtROS and ROS are responsible for improved bacterial clearance in trained larvae. To understand differences between *Shigella* and β-glucan training, we tested whether ROS production in zebrafish neutrophils is affected by β-glucan training (Fig. 6E and fig. S6H). In this case, we observed that β-glucan–trained neutrophils exposed to *Shigella* are not significantly depleted of ROS, as observed for *Shigella*-trained neutrophils (Fig. 6D). However, we found that β-glucan–trained neutrophils can sustain enhanced ROS production following *Shigella* infection, highlighting that host defense mechanisms used by trained neutrophils are different between *Shigella* and β-glucan training.

Our ChIP-seq dataset also pointed to activation of cellular senescence, particularly prosurvival and anti-apoptotic pathways (PI3K-Akt signaling and mitochondrial pathways; Fig. 5, C and E, and data files S2 and S3) were marked (*48*). To test whether *Shigella*-trained neutrophils could survive longer than naïve neutrophils, we used flow cytometry and found that there was a significant increase in the percentage of dead naïve neutrophils when exposed to *Shigella*, as compared to *Shigella*-trained neutrophils (Fig. 6F). These data support the hypothesis that trained neutrophils are more resilient to death upon *Shigella* reinfection.

To test whether trained immunity can affect other aspects of host defense, we investigated levels of myeloperoxidase (MPO) in trained neutrophils. Using the transgenic zebrafish line Tg(*mpx*::GFP) in which GFP expression is driven by the MPO promoter, we quantified MPO production using FACS. In agreement with its absence from ChIP-seq analysis, we did not observe significant differences in MPO production between naïve and trained neutrophils when reinfected (fig. S6I), highlighting the specificity of the *Shigella* training toward mtROS production. Together, these findings suggest that *Shigella*-trained neutrophils sustain H3K4me3 modifications that result in enhanced antimicrobial function via mtROS in preparation for a second challenge.

## DISCUSSION

Here, we introduce the use of zebrafish larvae to study innate immune training, demonstrating that local infection by *Shigella*, BCG, and β-glucan induces nonspecific protective effects. The zebrafish larval model is a highly attractive training model—as compared to in vitro (single-cell type system) and mouse models—as it is a vertebrate model with only innate immune responses during its first ~4 weeks of development.

Most work on trained immunity has been largely focused on monocytes and macrophages. However, there is growing evidence that neutrophils also play important roles upon training (*12*, *13*). In the case of *Shigella* infection, macrophages are unable to control infection as bacteria induce cell death programs (*31*, *33*, *49*). Although neutrophils can also be killed by *Shigella*, they resist for longer than macrophages and can rapidly eliminate bacteria (*31*, *33*). Here, we show that *Shigella* infection induces production of new neutrophils that are more protective and have better killing capacity than naïve neutrophils. We show that this enhanced infection control is linked to host control of its inflammatory state, which also contributes to increased survival upon a secondary infectious challenge. Considering that the immune status of zebrafish larvae returns to basal levels by 48 hp1i and that neutrophils retain epigenetic modifications following a first *Shigella* infection stimulus, we established that *Shigella* trains (and does not prime) the innate immune system of zebrafish. *Shigella* training protects against Gram-negative bacteria (as *Shigella* and *P. aeruginosa*) and also Gram-positive bacteria (as *S. aureus*), suggesting that it induces heterologous immunological memory. From our results showing that *Shigella* training increased survival and infection control, we conclude that the defense arm engaged by *Shigella* training contributes toward host resistance and not disease tolerance (*50*, *51*). Although we observed that expression of the anti-inflammatory cytokine IL-10 remained elevated by 48 hp1i, future work is required to identify the precise source of IL-10 and investigate whether *Shigella*-trained neutrophils can adopt an anti-inflammatory state (*52*).

To test the hypothesis that *Shigella* induces lasting training mechanisms in zebrafish larvae, we extended the interval between infections to 5 days and observed protection against *Shigella* in those larvae. Although protection levels might wane, this is in agreement with the decline of BCG-induced protection after vaccination (*53*, *54*). In the future, it will be of great interest to test adult zebrafish (>3 months) as well as investigate whether *Shigella* training mechanisms can be transmitted trans-generationally in zebrafish as shown using mice (*55*). We envision that with the practices of zebrafish laboratories to maintain adequate levels of heterozygosity (*56*), by consistently outbreeding their stocks (unlike mouse lines), the zebrafish model can be used to complement the mouse model aiming to elucidate transmission of trained immunity in humans.

Work in zebrafish has shown that BCG vaccination protects against *Mycobacterium marinum* and that β-glucan (systemic) protects against *Salmonella Typhimurium* (*24*, *27*). Both BCG and β-glucan training induce protection against *Shigella* infection in zebrafish but rely on distinct training pathways for whole animal infection control. Consistent with this, work using mice has shown that BCG induces training via nucleotide binding oligomerization domain containing 2 (NOD2) receptor and β-glucan via dectin-1 receptor (*38*, *39*, *57*). Although *Shigella* training phenotypes in zebrafish share similarities with those from BCG and β-glucan training (such as nonspecific protection and enhanced survival upon new infection), the H3K4me3-enriched pathways following *Shigella* training differ from those found in BCG-trained human neutrophils (*12*), which supports our conclusion that both stimuli induce different training mechanisms in neutrophils. Our findings suggest that *Shigella* training is multifactorial, depending on *Shigella* recognition by innate immune receptors, strong cytokine production, and an active T3SS.

Enriched pathways from our ChIP-seq datasets indicate that mtROS is playing an important role in enhancing antimicrobial activity of *Shigella*-trained neutrophils. Historically, ROS production in neutrophils has been associated with NADPH oxidase, and therefore, the bactericidal role of mtROS in neutrophils has remained mostly elusive. However, recent work in human and mouse neutrophils suggested that mitochondria generated ROS enhanced bactericidal activity (*58*, *59*). In agreement, our work highlights that mtROS in neutrophils plays a key role in bacterial killing, and we envision that future therapeutic strategies could manipulate mtROS by using pro-oxidants [such as SkQN (*60*) or MitoK3 (*61*)] specifically targeted to mitochondria in neutrophils.

Decades of research have tried to generate a vaccine with sufficient efficacy that could be used against different *Shigella* serotypes in humans (*62*). Our long-term goal is to guide vaccine studies in humans, exploiting innate immune memory to combat *Shigella* infections. A variety of animal models have significantly contributed to understanding shigellosis and feasibility of vaccines to combat it. We highlight zebrafish as an important animal model to investigate how innate immune training can be manipulated to control *Shigella* infection. It is next of great interest to train neutrophils using live *Shigella* in humans. In this way, we can discover fundamental components of immunity shared between zebrafish and humans that can be trained to promote a better immune response and combat *Shigella* infection.

## MATERIALS AND METHODS

### Ethics statements

Animal experiments were performed according to the Animals (Scientific Procedures) Act 1986 and approved by the Home Office (Project licenses: PPL P84A89400 and P4E664E3C).

### Zebrafish husbandry

Embryos were obtained from naturally spawning zebrafish, and larvae were maintained at 28.5°C in embryo medium (0.5× E2 medium), unless specified otherwise. Transgenic zebrafish lines used here were Tg(*lyz::*dsRed)^nz50^ (*63*), Tg(*mpx::*GFP)^i114^ (*64*), and Tg(*mpeg1*::Gal4-FF)^gl25^/Tg(UAS-E1b::*nfsB*.mCherry)^c264^ (*65*). For injections and live microscopy, larvae were anesthetized with tricaine (200 μg/ml; Sigma-Aldrich) in embryo medium. For injections in larvae >5 dpf, lidocaine (2 μg/liter) was used as an analgesic and added to the embryo medium [together with tricaine (200 μg/ml; Sigma-Aldrich)] before injection. After checking for full recovery from the anesthetic, larvae were kept in E2 medium with lidocaine (2 μg/liter) for 18 to 24 hours at 28.5°C. Larvae were monitored for appearance, righting reflex, reactive reflex, and opercular movement from day 5 dpf for two to three times a day. Larvae were not fed during the course of the experiment.

### Zebrafish injections

Bacterial strains used in this study can be found in table S1. *Shigella, Pseudomonas*, and *Staphylococcus* strains were grown on trypticase soy agar (TSA; Sigma-Aldrich) plates with the appropriate antibiotics—for *Shigella* growth, plates were supplemented with 0.01% Congo red (Sigma-Aldrich). Overnight cultures were grown from individual colonies at 37°C and 200 rpm, in 5 ml of trypticase soy broth (TSB; Sigma-Aldrich and Oxoid for *Staphylococcus*) supplemented with the appropriate antibiotics as above. For injections, bacteria were grown until an optical density (OD) of 0.55 to 0.65 at 600 nm (log phase) by diluting the overnight culture 50× in fresh TSB supplemented with the appropriate antibiotics. When in log phase, bacteria were spun down and washed in PBS (Sigma-Aldrich). The desired concentration was achieved by resuspension of bacteria in injection buffer [2% polyvinyl-pyrrolidone (PVP; Sigma-Aldrich) in PBS and 0.5% phenol red (Sigma-Aldrich)]. Control groups were injected with injection buffer (referred as PBS/naïve group).

For inactivated bacteria injections, suspensions were prepared as described above and, before injection, were incubated for 30 min at 60°C for heat killing or incubated in 4% PFA (Sigma-Aldrich) for 30 min at room temperature (RT) for PFA killing.

BCG was grown in 7H9 broth (supplemented with 10% albumin dextrose catalase (ADC) enrichment medium, 0.2% glycerol, and 0.02% Tween-80) until OD 0.5 to 0.6. The culture was then spun down and washed with PBS. The suspension was then centrifuged and resuspended in PVP to prevent large clumps. Single-cell suspensions were obtained by passing the bacteria through different gauge syringes (25G, 27G, and 29G) 10 times each. After achieving the desired concentration, bacteria were resuspended in injection buffer with higher concentration of PVP (4%).

A stock solution of β-glucan (Invivo Biosystems, catalog no. tlrl-bgp) was prepared at 20 mg/ml in PBS and stored at 4°C for a maximum of a month. For injections, indicated dilutions were prepared in injection buffer. Different amounts of β-glucan were first injected in the HBV of larvae to test for dose-dependent protection (fig. S3, C to E). The highest amount tested (20 μg/μl) was chosen as it suggested stronger protection responses.

LPS (Sigma-Aldrich, catalog no. L6143) was prepared to mimic the amount of bacteria injected in a nonlethal dose (2500 CFU). According to (*66*), 2500 bacterial cells in exponential phase would correspond to 0.125 ng of LPS. Therefore, a stock solution of LPS (0.25 mg/ml) in PBS was prepared and prior before injection was mixed 1:1 with 0.5% phenol red.

For injection, 1 to 2 nl of bacterial/β-glucan/LPS suspension or control solution was microinjected in the HBV of zebrafish larvae. For bacterial enumeration, larvae tissues were disrupted in 0.4% Triton X-100 (Sigma-Aldrich) with the aid of mechanical pestles, and serial dilutions were plated in the appropriate medium at the indicated time points. Plates were incubated at 37°C, and CFUs were enumerated when colonies were visible.

### Design of *S. flexneri* ∆*ospF* mutant

*S. flexneri* ∆*ospF* mutant was created using a λ-Red–mediated recombination (*67*). Briefly, pKD4 plasmid was used as template to amplify a kanamycin resistance–encoding DNA cassette using primers containing 50-bp nucleotides homologous to the site of insertion (table S1). *S. flexneri* electrocompetent cells producing λ-Red recombinase were electroporated with resulting polymerase chain reaction (PCR) fragments. Electroporated cells were then plated in TSA plates supplemented with 0.01% of Congo red and kanamycin (50 μg/ml). To remove the kanamycin resistance–encoding DNA cassette, bacteria were transformed with pCP20 plasmid [that encodes the yeast *flp* recombinase (*68*)]. Deletions were verified by PCR using confirmation primers in table S1.

### Microscopy and image analysis

Whole larvae and HBV images were acquired using stereo fluorescent microscope Leica M205FA (Leica, Germany) and by placing anesthetized larvae on 1% agarose-E2 plates.

Image files were processed using ImageJ/Fiji software. Whole larvae leukocyte quantification was performed according to (*69*) with modifications. Briefly, fluorescent images were converted to binary in ImageJ/Fiji, resulting in images in which fluorescence was converted into black pixels onto a white background. For each image, pixels of five individual cells were quantified. Total pixel count of each larva was divided by the average of five individual leukocytes to determine the total number of cells. Leukocyte quantification in the HBV was determined manually in the region highlighted in Fig. 1G.

### Zebrafish chemical treatments

For inflammasome stimulation, larvae were kept at 28.5°C in E2 medium with 0.1 μM nigericin (Sigma-Aldrich, catalog no. 481990-5MG) or 1% d-glucose (Sigma-Aldrich, catalog no. G8270-100G) (*18*) for 24, 48, or 96 hours from 2 dpf. For NAPDH ROS and mtROS inhibition after the second infection, larvae were kept at 28.5°C in E2 medium with 100 μM DPI (Sigma-Aldrich, catalog no. D2926) (*70*) and 10 μM CsA (Enzo Life Sciences, catalog no. BML-A195-0100) (*71*), both in dimethyl sulfoxide (DMSO), for 6 hours until larvae were dissociated for flow cytometry. Control larvae were kept in E2 with 1% DMSO.

### RNA extraction, cDNA synthesis, and quantitative reverse transcription–PCR

RNA was extracted from 5 to 10 snap-frozen larvae with the RNeasy Mini kit (Qiagen, catalog no. 74104) and reverse-transcribed using QuantiTect Reverse Transcription kit (Qiagen, catalog no 205311) according to manufacturer’s instructions. Quantitative PCR (qPCR) was performed using the 7500 Fast Real-Time PCR System machine and 7500 Fast Real-Time PCR software v2.3 (Applied Biosystems, Foster City, CA) and SYBR green master mix (Applied Biosystems, catalog no. 10187094). Template cDNA was subjected to PCR using primers described in table S1, and samples were run in technical duplicates. The comparative Ct method was used for gene expression quantification, and *ef1a1l1* was used as the housekeeping gene.

### Flow cytometry

Tg(*lyz::*dsRed)^nz50^ and Tg(*mpx::*GFP)^i114^ transgenic larvae were dissociated for flow cytometry following the protocol from (*72*), with some modifications. Briefly, 20 to 100 larvae were washed twice in calcium-free Ringer’s solution for 10 min with slow shaking at RT. After removal of Ringer’s solution, 2 ml of dissociation solution [0.5% Trypsin EDTA 10× no phenol (Thermo Fisher Scientific, catalog no.15400054)] was added. Larvae were incubated twice for 10 min at 28.5°C, with gentle pipetting in between incubations to help tissue digestion. The reaction was stopped by adding 10% fetal calf serum (Themo Fisher Scientific, catalog no. 11550356). The suspensions were sieved using 70-μm Nylon cell strainers (Merck, CLS431751-50EA) or cell strainer snap caps from test tubes (Corning, 352235) and then gently centrifuged for 3 min at 800*g*. Cells were then washed with 1 ml of PBS with 10 mM Hepes and 1 mM EDTA. For live/dead staining, cells were incubated with either LIVE/DEAD fixable near IR dead cell stain (1:2000; Thermo Fisher Scientific, catalog no. L34994) or LIVE/DEAD fixable violet dead cell stain (1:2000; Thermo Fisher Scientific, catalog no. L34964) dyes for 30 min at 28.5°C. Cells were fixed with 4% PFA for 10 min at RT for flow cytometry. For ROS staining, 5 μM CellROX Deep Red (Thermo Fisher Scientific, catalog no. C10422) was added to the E2 medium for 30 min before larvae dissociation, and the larvae were kept in the dark at 28.5°C. Single cells were measured on a LSRII (BD Biosciences) or on an Attune NxT Flow Cytometer (Thermo Fisher Scientific). For mtROS staining, 300 nM MitoTracker Red CM-H_2_XRos (Thermo Fisher Scientific, catalog no. M7513) was added to the dissociated cells for 30 min at 28.5°C. Single cells were measured on a FACSAria III cell sorter (BD Biosciences). Data were analyzed with FlowJo software v10.7.1. To prepare samples for ChIP-seq, cells were fixed with 1% formaldehyde for 8 min at RT with occasional mixing. To stop the reaction, 100 μl of glycine 1.25 M was added, and the samples were incubated for 5 min at RT. After centrifuging at 600*g* for 10 min at 4°C, cells were washed with ice-cold PBS containing protease inhibitor cocktail (Sigma-Aldrich, catalog no. 5892970001) and centrifuged again. Cells were then resuspended in PBS with 10 mM Hepes and 1 mM EDTA. FACS sorting was done on a FACSAria III cell sorter (BD Biosciences). Data were analyzed with BD FACSDiva software. Gating strategies can be found in fig. S5A for neutrophil sorting, in fig. S6A for mtROS measurement, and fig. S6E for CellROX measurement.

### ChIP-seq and analysis

For ChIP experiments, a minimum of 20,000 sorted Tg(*lyz*:dsRed) neutrophils were collected per sample (PBS, *Shigella*, and *Shigella* ∆T3SS) and per replicate (each biological replicate corresponds to two experiments). Cells were kept at –80°C until sequencing. ChIP-seq was conducted by Diagenode ChIP-seq/ChIP-qPCR Profiling service (Diagenode, catalog no. G02010000). Chromatin was prepared for ChIP-seq using the True MicroChIP Kit (Diagenode, catalog no. C01010130) and was sheared using Bioruptor Pico sonication device (Diagenode, catalog no. B01060001) combined with the Bioruptor Water cooler for 5 cycles using a 30″ (on) 30″ (off) settings. Optimization of shearing conditions was done on chromatin corresponding to 10,000 cells. Immunoprecipitation was done on chromatin corresponding to the remaining 10,000 cells using antibodies against H3K4me3 (Diagenode, catalog no. C15410003)—10% of the chromatin was used for input. Libraries were prepared using the IP-Star Compact Automated System (Diagenode, catalog no. B03000002) from input and ChIP’d DNA using MicroPlex Library Preparation Kit v2 (12 indices) (Diagenode, catalog no. C05010013). Libraries were pooled and sequenced with Illumina Novaseq 6000, running NovaSeq Control Software 1.6.0, with paired-end reads of 50-bp length. Quality control of sequencing reads was performed using FastQC (*73*).

ChIP-seq reads were analyzed using the Galaxy platform (usegalaxy.eu). Reads were aligned to the reference genome GRCz10 using the Bowtie2 software (*74*), and PCR duplicates were removed with MarkDuplicates (*75*). Peak calling was performed using MACS2 callpeak (*76*), and the narrow peaks lists were used in Irreproducible Discovery Rate (IDR) (*77*) to identify reproducible peaks between replicates. Last, ChIPseeker (*78*) was used for peak annotation with a promoter defined as ±3 kb around the transcription start site (TSS). IGV v2.9.4 (*79*) was used for peak visualization.

For pathway annotation, the lists of genes associated with promoter regions highlighted in bold in Fig. 5 (B and D) and fig. S5 (B and D) (PBS, *Shigella* WT, and *Shigella* ΔT3SS) were analyzed against Kyoto Encyclopedia of Genes and Genomes (KEGG) pathways in Cytoscape v3.9.1 (*80*) using the plug-in ClueGO v2.5.9 (*81*).

### Statistical analysis

Statistical significance was determined using GraphPad Prism v9. For differences in survival curves, the log-rank (Mantel-Cox) test was used. Data from bacterial burden and gene expression levels were log_10_- or log_2_-transformed, respectively. Pairwise comparisons were determined using a Student’s unpaired *t* test. For multiple comparisons, one-way or two-way analysis of variance (ANOVA) tests with Sidak’s or Tukey’s corrections were used, as indicated in the figure legend.
