# Supplementary material for: Shigella induces epigenetic reprogramming of zebrafish neutrophils
Source: Sci Adv. 2023 Sep 6;9(36):eadf9706. doi: 10.1126/sciadv.adf9706 (PMC10482349; doi:10.1126/sciadv.adf9706)
Supplement: Supplementary file 1 — Table S1 Figs. S1 to S6 Legends for data files S1 to S3 [file sciadv.adf9706_sm.pdf]

Science Advances
AAAS

Supplementary Materials for

Shigella induces epigenetic reprogramming of zebrafish neutrophils

Margarida C. Gomes *et al.*

Corresponding author: Margarida C. Gomes, margarida.castro-gomes@lshtm.ac.uk;
Serge Mostowy, serge.mostowy@lshtm.ac.uk

Sci. Adv. **9**, eadf9706 (2023)
DOI: 10.1126/sciadv.adf9706

The PDF file includes:

Table S1
Figs. S1 to S6
Legends for data files S1 to S3

Other Supplementary Material for this manuscript includes the following:

Data files S1 to S3

SUPPLEMENTAL TABLES

Table S1. Bacterial strains, qPCR primers and primers for *ospF* mutation in *S. flexneri* used in this study

Bacterial strains				
**Species**	**Genotype**	**Reporter**	**Resistance**	**Reference**
Shigella flexneri M90T	WT	GFP	Carbenicillin	(*96*)
Shigella flexneri M90T	WT	mCherry	Carbenicillin	(*45*)
Shigella flexneri M90T	WT	Ruby	Carbenicillin	(*97*)
Shigella flexneri M90T	Δ*mxiD*	GFP	Carbenicillin	This study
Shigella flexneri M90T	Δ*rfaC*	GFP	Carbenicillin	(*56*)
Shigella flexneri M90T	Δ*mxiE*	Ruby	Carbenicillin	(*97*)
Shigella flexneri M90T	Δ*ospF*		Kanamycin	This study
Pseudomonas aeruginosa PA14	WT	GFP (chromosomal)	Gentamycin	(*98*)
Staphylococcus aureus RN6390	WT	GFP (chromosomal)		(*99*)
Mycobacterium bovis BCG (Russia)	WT	GFP	Kanamycin	(*100*)

qPCR primers			
**Name**	**GeneID**	**Sequence (5'-3')**	**Reference**
*eef1a1a*FW	ENSDARG00000039502	AAGCTTGAAGACAACCCCA AGAGC	(*101*)
*eef1a1a*RV	ENSDARG00000039502	ACTCCTTTAATCACTCCCAC CGCA	(*101*)
*cxcl8a*FW	ENSDARG00000104795	TGTGTTATTGTTTTCCTGGC ATTTC	(*102*)
*cxcl8a*RV	ENSDARG00000104795	GCGACAGCGTGGATCTACA G	(*102*)
*cxcl18b*FW	ENSDARG00000075045	TCTTCTGCTGCTGCTTGCG GT	(*103*)
*cxcl18b*RV	ENSDARG00000075045	GGTGTCCCTGCGAGCACGA T	(*103*)
*il1b*FW	ENSDARG00000098700	GAACAGAATGAAGCACATC AAACC	(*103*)
*il1b*RV	ENSDARG00000098700	ACGGCACTGAATCCACCAC	(*103*)
*il6*FW	ENSDARG00000102318	TCAACTTCTCCAGCGTGATG	(*104*)
*il6*RV	ENSDARG00000102318	TCTTTCCCTCTTTTCCTCCT G	(*104*)
*il10*FW	ENSDARG00000078147	CATAACATAAACAGTCCCTA TG	(*105*)
*il10*RV	ENSDARG00000078147	GTACCTCTTG CATTTCACCA	(*105*)
*tnfa*FW	ENSDARG00000009511	AGACCTTAGACTGGAGAGA TGAC	(*102*)
*tnfa*RV	ENSDARG00000009511	CAAAGACACCTGGCTGTAG AC	(*102*)

*gscfa*FW	ENSDARG00000102211	GCTTTTTGATTGGTGTTGCTATAATG	(*29*)
*gcsfa*RV	ENSDARG00000102211	CAACGATCCCCACTAATGTGAA	(*29*)
*mmp9*FW	ENSDARG00000042816	CATTAAAGATGCCCTGATGTATCCC	(*102*)
*mmp9*RV	ENSDARG00000042816	AGTGGTGGTCCGTGGTTGAG	(*102*)

Primers for *ospF* mutation in *S. flexneri*

**Name**	**Sequence (5'-3')**	**Notes**
OspFMutFw	GAAGCAGCTCCAGCCTACACAACAGGGCTTTTTTATGGGCATAGAAAACGTCCTCTATAAAATAGATATA	Deletion *ospF*
OspFMutRv	CTAAGGAGGATATTCATATGTATCGTTTGATGATAGAGTAGAGAAGATGCCAGCTACACCACCTTGTAGC	Deletion *ospF*
OspF_Conf5	AAAAGATGAAGGCCTGATGGGAGCATTAAC	Confirmation of *ospF* deletion
OspF_Conf3	TGGTGGATAAAACCCGCCAGAATGAACA	Confirmation of *ospF* deletion

SUPPLEMENTAL FIGURES

Figure S1

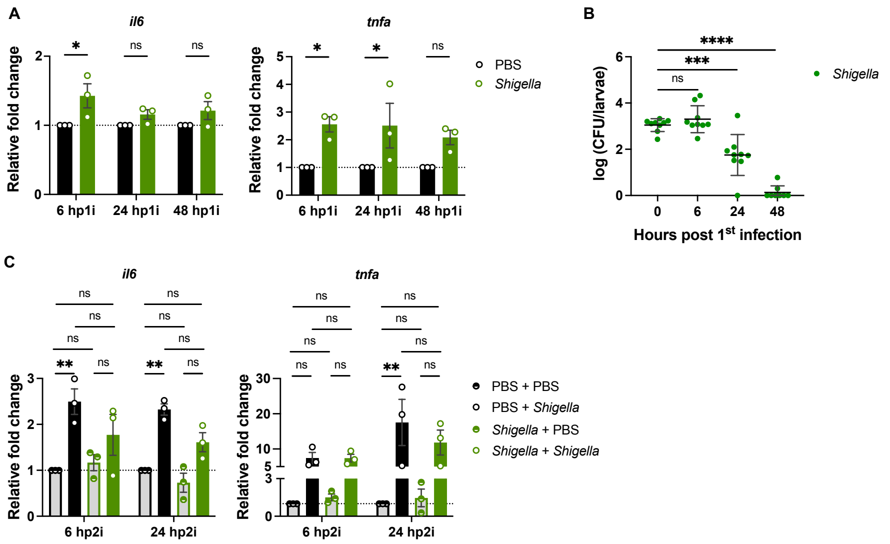

Figure S1, related to Figure 1 – Expression of *il6* and *tnfa* during training and reinfection.

(A) Fold change in the expression of *il6* and *tnfa* in larvae injected with non-lethal dose of *S. flexneri* M90T (green full bars, $1.3x10^3 \pm 5.5x10^2$ CFUs) as compared to PBS control (naïve-black full bars). Data pooled from 3 experiments with n>5 larvae per time point per condition per experiment (mean ± SD, *p < 0.05, 2-way ANOVA with Sidak's multiple comparisons test).

(B) Log_{10}-transformed CFU counts *Shigella*-trained larvae injected with a non-lethal dose of *S. flexneri* M90T ($1.3x10^3 \pm 5.5x10^2$ CFUs). Data pooled from 3 independent experiments using n=3 larvae per condition per experiment (mean ± SD, ***p < 0.001, ****p < 0.0001, 1-way ANOVA with Dunnett's multiple comparisons test).

(C) Fold change in the expression of *il6* and *tnfa* following lethal dose injection of *S. flexneri* M90T in naïve (black full bars, $2.5x10^4 \pm 1x10^4$ CFUs) and *Shigella*-trained (green full bars, $2.7x10^4 \pm 4.5x10^3$ CFUs) as compared to PBS injected controls (grey bars). Data pooled from 3 experiments with n>5 larvae per time point per condition per experiment (mean ± SD, **p < 0.01, 2-way ANOVA with Sidak's multiple comparisons test).

Figure S2

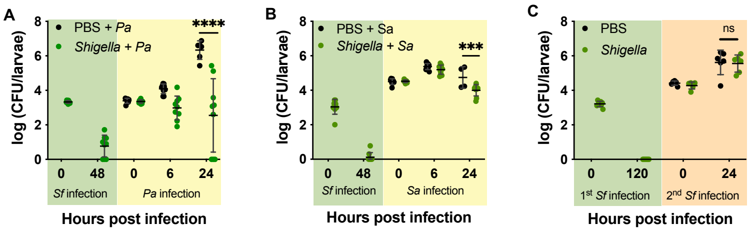

Figure S2, related to Figure 2 – Bacterial burden during reinfection with *P. aeruginosa* and *S. aureus*, and after 5 dp1i.

(A) Log$_{10}$-transformed CFU counts of naïve and *Shigella*-trained (in green section: 2.2x10^{3} ± 3.8x10^{2} CFUs) larvae injected with a lethal dose of *P. aeruginosa* (in orange section: PBS – 2.6x10^{3} ± 7.4x10^{2} CFUs, *Shigella* – 2.3x10^{3} ± 5.5x10^{2} CFUs). Data pooled from 3 independent experiments using n=3 larvae per condition per experiment (mean ± SD, ****$p < 0.0001$, 1-way ANOVA with Tukey's multiple comparisons test).

(B) Log$_{10}$-transformed CFU counts of naïve and *Shigella*-trained (in green section: 1.4x10^{3} ± 8.6x10^{2} CFUs) larvae injected with a non-lethal dose of *S. aureus* (PBS – 3.4x10^{4} ± 1.1x10^{4} CFUs, *Shigella* – 3.4x10^{4} ± 6x10^{3} CFUs). Data pooled from 3 independent experiments using n=3 larvae per condition per experiment (mean ± SD, 1-way ANOVA with Tukey's multiple comparisons test).

(C) Log$_{10}$-transformed CFU counts of naïve and *Shigella*-trained (in green section: 1.7x10^{3} ± 5.7x10^{2} CFUs) larvae injected with a lethal dose of *S. flexneri* M90T (in red section: PBS - 2.7x10^{4} ± 4.5x10^{3} CFUs, *Shigella* - 2.5x10^{4} ± 1x10^{4} CFUs). Data pooled from 3 independent experiments using n>2 larvae per condition per experiment (mean ± SD, 1-way ANOVA with Tukey's multiple comparisons test).

Figure S3

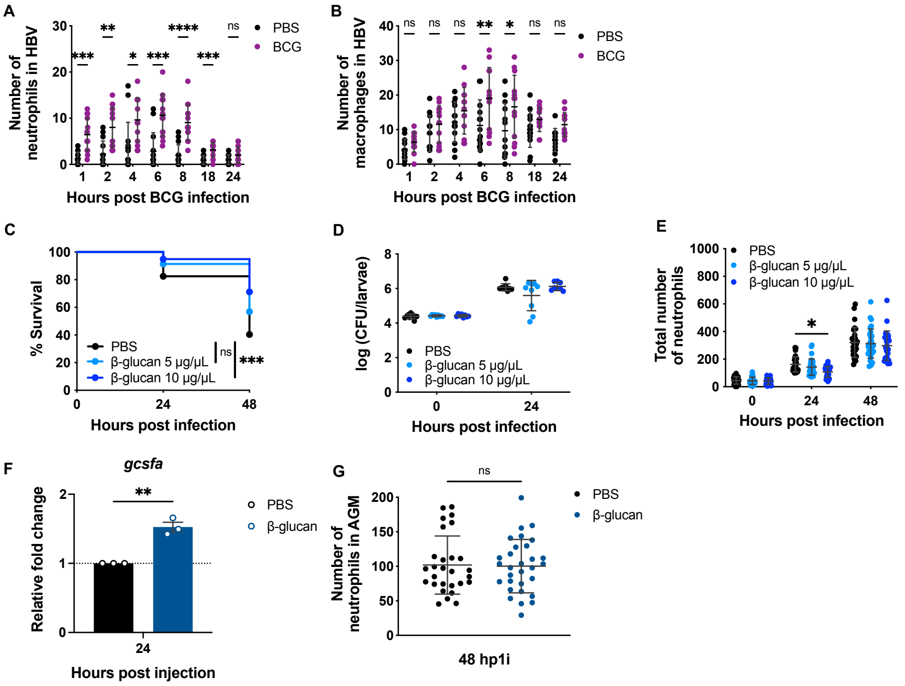

Figure S3, related to Figure 3 – Leukocyte recruitment to BCG infected HBV, testing different β-glucan concentrations, and granulopoiesis in β-glucan training.

(A) Quantification of recruited neutrophils (Tg(*lyz*::dsRed)) to the HBV in naïve (black full circles) and BCG-trained (purple full circles, $4x10^2 \pm 1.5x10^2$ CFUs) larvae. Data pooled from 3 experiments with n>4 larvae per time point per condition per experiment (mean ± SD, 2-way ANOVA with Sidak's multiple comparisons test).

(B) Quantification of recruited macrophages (Tg(*mpeg*::mCherry)) to the HBV in naïve (black full circles) and BCG-trained (purple full circles, $4x10^2 \pm 1.5x10^2$ CFUs) larvae. Data pooled from 3 experiments with n>4 larvae per time point per condition per experiment (mean ± SD, 2-way ANOVA with Sidak's multiple comparisons test).

(C) Survival curves from naïve (black full circles) and β-glucan-trained (light blue full circles: 5 ng, dark blue full circles: 10 ng) larvae infected with a lethal dose of *S. flexneri* M90T. Data pooled from 3 independent experiments using n>18 larvae per condition per experiment (**p < 0.01, Log-rank (Mantel-Cox) test).

(D) Log_{10}-transformed CFU counts of naïve and β-glucan-trained larvae injected with a lethal dose of *S. flexneri* M90T (PBS – $2.5x10^4 \pm 7.3x10^3$ CFUs, β-glucan 5 ng – $2.7x10^4 \pm 3.2x10^3$ CFUs, β-glucan 10 ng– $2.7x10^4 \pm 5.1x10^3$ CFUs). Data pooled from 3 independent experiments using n=3 larvae per condition per experiment (mean ± SD, 2-way ANOVA with Sidak's multiple comparisons test).

(E) Quantification of neutrophils (Tg(*lyz*::dsRed)) at 0, 24 and 48 hp1i injected with PBS (naïve- black full circles) and a non-lethal dose of β-glucan (light blue full circles: 5 ng, dark blue full circles: 10 ng). Data pooled from 3 experiments with n>8 larvae per time point per condition per experiment (mean ± SD, *p < 0.05, 2-way ANOVA with Sidak's multiple comparisons test).

(F) Fold change in the expression of *gcsfa* following non-lethal dose injection of β-glucan (blue bars, 20 ng) as compared to PBS injected controls (black bars). Data pooled from 3

experiments with n=10 larvae per time point per condition per experiment (mean ± SD, **p < 0.01, unpaired Student's *t*-test).

(G) Quantification of neutrophils (Tg(*lyz*::dsRed)) in the AGM at 48 hp1i injected with PBS (naïve- black full circles) and a non-lethal dose of β-glucan (blue full circles, 20 ng). Data pooled from 3 experiments with n>8 larvae per time point per condition per experiment (mean ± SD, unpaired Student's *t*-test).

Figure S4

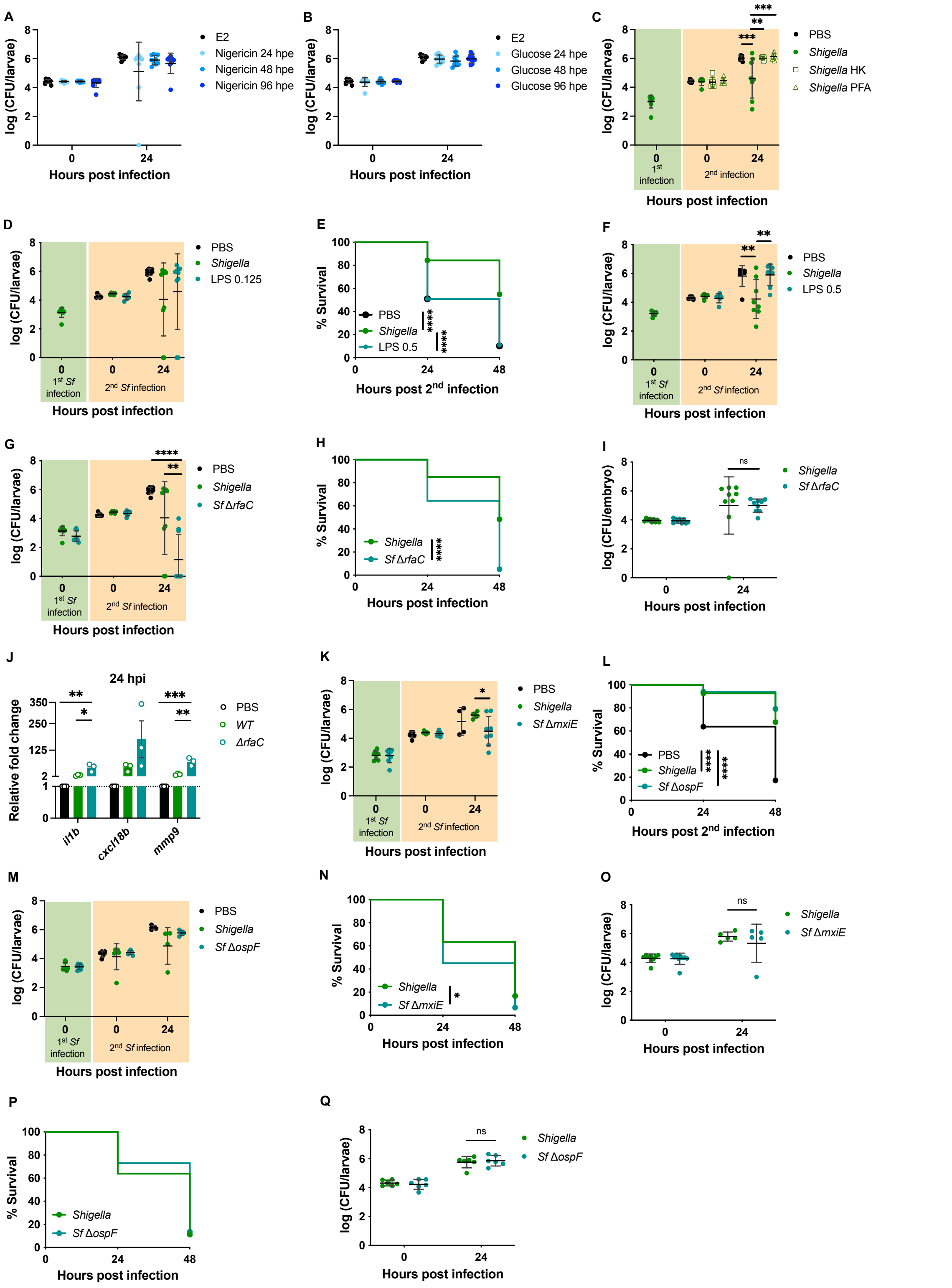

Figure S4, related to Figure 4 – Bacterial burden during reinfection upon stimulation of inflammatory pathways and virulence assays with *Shigella* mutants.

(A) Log_{10}-transformed CFU counts from E2 (embryo media control, black full circles) and nigericin (blue full circles, 0.1 µM) treated larvae infected with a lethal dose of *S. flexneri* M90T (PBS – $2.7x10^4$ ± $8.5x10^3$ CFUs, nigericin – $2.6x10^4$ ± $4.1x10^3$ CFUs). Data pooled from 3 independent experiments using n=3 larvae per condition per experiment (mean ± SD, 2-way ANOVA with Sidak's multiple comparisons test).

(B) Log_{10}-transformed CFU counts from E2 (embryo media control, black full circles) and glucose (blue full circles, 1%) treated larvae infected with a lethal dose of *S. flexneri* M90T (PBS – $2.7x10^4$ ± $8.5x10^3$ CFUs, glucose – $2.7x10^4$ ± $1x10^4$ CFUs). Data pooled from 3 independent experiments using n=3 larvae per condition per experiment (mean ± SD, 2-way ANOVA with Sidak's multiple comparisons test).

(C) Log_{10}-transformed CFU counts from naïve (black full circles), live *Shigella*-trained (green full circles, $1.3x10^3$ ± $6.6x10^2$ CFUs), heat-killed (HK) *Shigella*-trained (dark green/white squares) and PFA-killed *Shigella*-trained (dark green/white triangles) larvae infected with a lethal dose of *S. flexneri* M90T (PBS – $2.7x10^4$ ± $6.1x10^3$ CFUs, *Shigella* – $2.7x10^4$ ± $1x10^4$ CFUs, *Shigella* HK – $3.2x10^4$ ± $3.3x10^4$ CFUs, *Shigella* PFA – $3.2x10^4$ ± $1.7x10^4$ CFUs). Data pooled from 3 independent experiments using n=3 larvae per condition per experiment (mean ± SD, **p < 0.01, ****p < 0.0001, 2-way ANOVA with Sidak's multiple comparisons test).

(D) Log_{10}-transformed CFU counts from naïve (black full circles), *Shigella*-trained (green full circles, $1.6x10^3$ ± $6.5x10^2$ CFUs) and LPS-primed (moss green full circles, 0.125 ng) larvae infected with a lethal dose of *S. flexneri* M90T (PBS – $1.9x10^4$ ± $6.3x10^3$ CFUs, *Shigella* – $2.8x10^4$ ± $4.7x10^3$ CFUs, LPS – $1.9x10^4$ ± $9.1x10^3$ CFUs). Data pooled from 3 independent experiments using n=3 larvae per condition per experiment (mean ± SD, 2-way ANOVA with Sidak's multiple comparisons test).

(E) Survival curves from naïve (black full circles), *Shigella*-trained (green full circles, $1.7x10^3$ ± $5.5x10^2$ CFUs) and LPS-primed (moss green full circles, 0.5 ng) larvae infected with a lethal dose of *S. flexneri* M90T (PBS – $2x10^4$ ± $4.6x10^3$ CFUs, *Shigella* – $2.7x10^4$ ± $8.2x10^3$ CFUs, LPS – $2.3x10^4$ ± $1.2x10^4$ CFUs). Data pooled from 3 independent experiments using n>8 larvae per condition per experiment. (****p < 0.0001, Log-rank (Mantel-Cox) test).

(F) Log_{10}-transformed CFU counts from naïve (black full circles), *Shigella*-trained (green full circles, $1.7x10^3$ ± $5.5x10^2$ CFUs) and LPS-primed (moss green full circles, 0.5 ng) larvae infected with a lethal dose of *S. flexneri* M90T (PBS – $2x10^4$ ± $4.6x10^3$ CFUs, *Shigella* – $2.7x10^4$ ± $8.2x10^3$ CFUs, LPS – $2.3x10^4$ ± $1.2x10^4$ CFUs). Data pooled from 3 independent experiments using n=3 larvae per condition per experiment (mean ± SD, **p < 0.01, 2-way ANOVA with Sidak's multiple comparisons test).

(G) Log_{10}-transformed CFU counts from naïve (black full circles), *Shigella*-trained (green full circles, $1.6x10^3$ ± $6.5x10^2$ CFUs) and Shigella Δ*rfaC*-trained (moss green full circles, $8.5x10^2$ ± $7.5x10^2$ CFUs) larvae infected with a lethal dose of *S. flexneri* M90T (PBS – $1.9x10^4$ ± $6.3x10^3$ CFUs, *Shigella* – $2.8x10^4$ ± $4.7x10^3$ CFUs, Δ*rfaC* – $2.4x10^4$ ± $8.4x10^3$ CFUs). Data pooled from 3 independent experiments using n=3 larvae per condition per experiment (mean ± SD, **p < 0.01, 2-way ANOVA with Sidak's multiple comparisons test).

(H) Survival curves from virulence assay of 3 dpf larvae infected with *Shigella* (green full circles, $9.6x10^3$ ± $2.5x10^3$ CFUs) and *Shigella* Δ*rfaC* (moss green full circles, $9.1x10^3$ ± $3.2x10^2$ CFUs). Data pooled from 3 independent experiments using n>8 larvae per condition per experiment. (****p < 0.0001, Log-rank (Mantel-Cox) test).

(I) Log_{10}-transformed CFU counts from virulence assay of 3 dpf larvae infected with *Shigella* (green full circles, $9.6x10^3$ ± $2.5x10^3$ CFUs) and *Shigella* Δ*rfaC* (moss green full circles,

$9.1x10^3 \pm 3.2x10^2$ CFUs). Data pooled from 3 independent experiments using n=3 larvae per condition per experiment (mean ± SD, 1-way ANOVA with Tukey's multiple comparisons test).

(J) Fold change in the expression of *il1b, cxcl18b* and *mmp9* following injection of PBS (black full bars), *Shigella* (green full bars, $9.6x10^3 \pm 2.5x10^3$ CFUs) and *Shigella* Δ*rfaC* (moss green full bars, $9.1x10^3 \pm 3.2x10^2$ CFUs). Data pooled from 3 experiments with n>5 larvae per time point per condition per experiment (mean ± SEM, *p < 0.05, **p < 0.01, ***p < 0.001, 2-way ANOVA with Sidak's multiple comparisons test).

(K) Log_{10}-transformed CFU counts from naïve (black full circles), *Shigella*-trained (green full circles, $7.9x10^2 \pm 5.5x10^2$ CFUs) and Shigella Δ*mxiE*-trained (moss green full circles, $8.8x10^2 \pm 5.8x10^2$ CFUs) larvae infected with a lethal dose of *S. flexneri* M90T (PBS – $1.9x10^4 \pm 7.5x10^3$ CFUs, *Shigella* – $2.5x10^4 \pm 4.6x10^3$ CFUs, Δ*mxiE* – $2.2x10^4 \pm 7.6x10^3$ CFUs). Data pooled from 3 independent experiments using n=3 larvae per condition per experiment (mean ± SD, 2-way ANOVA with Sidak's multiple comparisons test).

(L) Survival curves from naïve (black full circles), *Shigella*-trained (green full circles, $3.4x10^3 \pm 2.2x10^3$ CFUs) and *Shigella* Δ*ospF*-trained (moss green full circles, $2.9x10^3 \pm 1.1x10^3$ CFUs) larvae infected with a lethal dose of *S. flexneri* M90T (PBS – $2.4x10^4 \pm 8.5x10^3$ CFUs, *Shigella* – $2.7x10^4 \pm 1.5x10^3$ CFUs, Δ*ospF* – $2.8x10^4 \pm 8.9x10^3$ CFUs). Data pooled from 3 independent experiments using n>10 larvae per condition per experiment. (****p < 0.0001, Log-rank (Mantel-Cox) test).

(M) Log_{10}-transformed CFU counts from naïve (black full circles), *Shigella*-trained (green full circles, $3.4x10^3 \pm 2.2x10^3$ CFUs) and *Shigella* Δ*ospF*-trained (moss green full circles, $2.9x10^3 \pm 1.1x10^3$ CFUs) larvae infected with a lethal dose of *S. flexneri* M90T (PBS – $2.4x10^4 \pm 8.5x10^3$ CFUs, *Shigella* – $2.7x10^4 \pm 1.5x10^3$ CFUs, Δ*ospF* – $2.8x10^4 \pm 8.9x10^3$ CFUs). Data pooled from 3 independent experiments using n=3 larvae per condition per experiment (mean ± SD, *p < 0.05, 2-way ANOVA with Sidak's multiple comparisons test).

(N) Survival curves from virulence assay of 3 dpf larvae infected with *Shigella* (green full circles, $2.3x10^4 \pm 9.6x10^3$ CFUs) and *Shigella* Δ*mxiE* (moss green full circles, $2.2x10^4 \pm 9.8x10^3$ CFUs). Data pooled from 3 independent experiments using n=20 larvae per condition per experiment. (*p < 0.05, Log-rank (Mantel-Cox) test).

(O) Log_{10}-transformed CFU counts from virulence assay of 3 dpf larvae infected with *Shigella* (green full circles, $2.3x10^4 \pm 9.6x10^3$ CFUs) and *Shigella* Δ*mxiE* (moss green full circles, $2.2x10^4 \pm 9.8x10^3$ CFUs). Data pooled from 3 independent experiments using n=3 larvae per condition per experiment (mean ± SD, 1-way ANOVA with Tukey's multiple comparisons test).

(P) Survival curves from virulence assay of 3 dpf larvae infected with *Shigella* (green full circles, $2.3x10^4 \pm 1.1x10^3$ CFUs) and *Shigella* Δ*ospF* (moss green full circles, $2.1x10^4 \pm 1.3x10^4$ CFUs). Data pooled from 2 independent experiments using n>15 larvae per condition per experiment. (*p < 0.05, Log-rank (Mantel-Cox) test).

(Q) Log_{10}-transformed CFU counts from virulence assay of 3 dpf larvae infected with *Shigella* (green full circles, $2.3x10^4 \pm 1.1x10^3$ CFUs) and *Shigella* Δ*ospF* (moss green full circles, $2.1x10^4 \pm 1.3x10^4$ CFUs). Data pooled from 2 independent experiments using n=3 larvae per condition per experiment (mean ± SD, 1-way ANOVA with Tukey's multiple comparisons test).

Figure S5

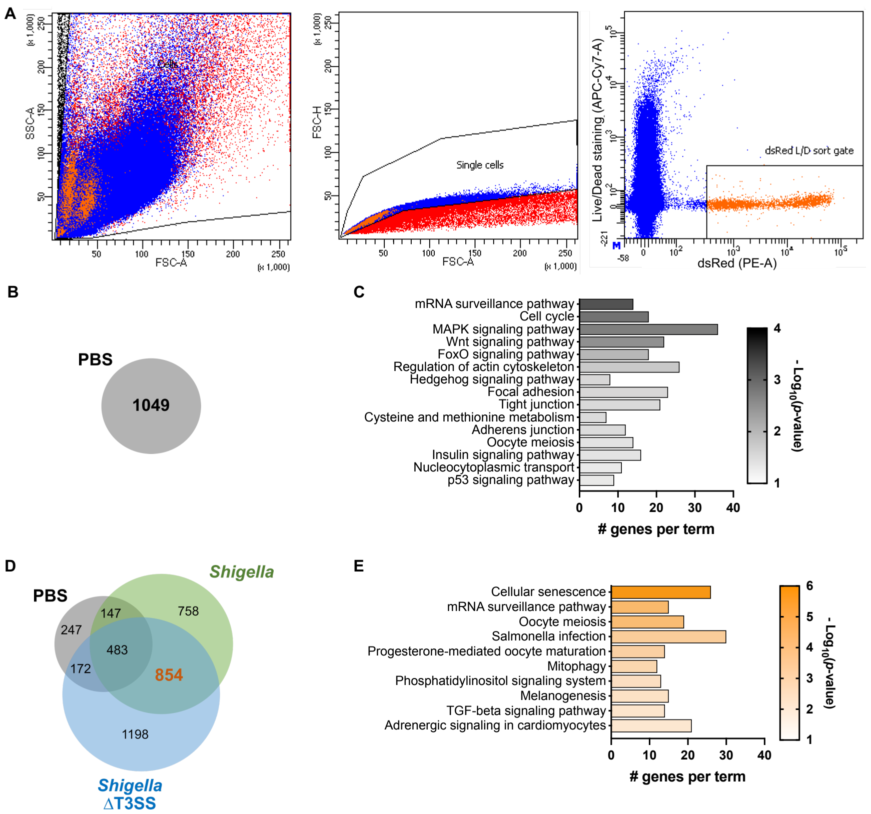

Figure S5, related to Figure 5 – ChIP-seq analysis of PBS, *Shigella*- and *Shigella* ΔT3SS-trained neutrophils.

(A) Gating strategy for FACS sorting neutrophils from Tg(*lyz*::dsRed) larvae. Post cell identification, duplet exclusion and determination of live and dead cells (LIVE/DEAD^{TM} Fixable Near IR Stain), neutrophils were identified by dsRed expression, followed by single cell sorting.

(B) Venn diagram showing number of genes marked by H3K4me3 peaks in their promoter regions (±3kb from TSS) of PBS treated larvae.

(C) Enriched KEGG-pathways (*p*-value < 0.01) associated with the 1049 genes marked by a H3K4me3 peak in their promoter regions (±3kb from TSS) in PBS treated larvae.

(D) Venn diagram showing number of common and unique genes marked by H3K4me3 peaks in their promoter regions (±3kb from TSS) of PBS, *Shigella* WT and *Shigella* ΔT3SS trained larvae.

(E) Enriched KEGG-pathways (*p*-value < 0.01) associated with the 854 genes marked by a H3K4me3 peak in their promoter regions (±3kb from TSS) common to *Shigella* WT and *Shigella* ΔT3SS trained larvae.

Figure S6

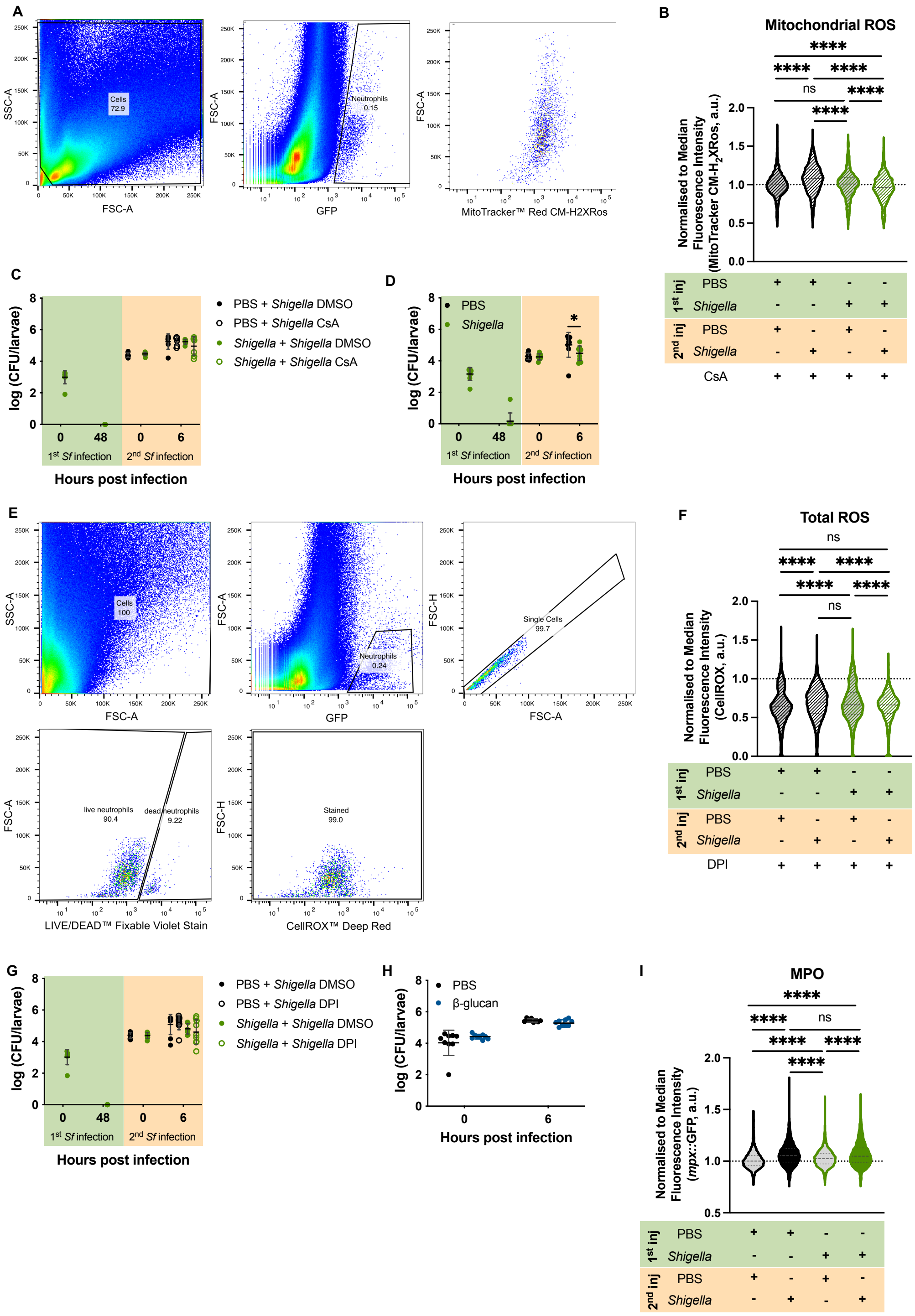

Figure S6, related to Figure 6 – Quantifying ROS in trained neutrophils.
(A) Gating strategy for flow cytometry of neutrophils from Tg(*mpx*::GFP) larvae. Neutrophils were identified by *mpx-gfp* expression. Neutrophils were analysed for their mitochondrial ROS production by fluorescence intensity detection of MitoTracker Red CM-H2Xros.

(B) Normalised fluorescence of MitoTracker Red CM-H_2XRos dye neutrophils treated with CsA from naïve (black outlines) and *Shigella*-trained (green outlines, $1.2x10^3 \pm 5.3x10^2$ CFUs) larvae (Tg(*mpx*::GFP)) unstimulated (grey fills) or infected with a lethal dose of *S. flexneri* M90T (PBS – black fill, $2.6x10^4 \pm 9.3x10^3$ CFUs, *Shigella* – green fill, $3x10^4 \pm 6.6x10^3$ CFUs)

at 6 hp2i. Data pooled from 3 experiments with n>10 larvae per condition per experiment (mean ± SD, ****p < 0.0001, 1-way ANOVA with Tukey's multiple comparisons test). a.u.: arbitrary units.

(C) Log_{10}-transformed CFU counts of naïve and *Shigella*-trained ($1.2x10^3 \pm 5.3x10^2$ CFUs) larvae injected with a lethal dose of *S. flexneri* M90T (PBS – black fill, $2.6x10^4 \pm 9.3x10^3$ CFUs, *Shigella* – green fill, $3x10^4 \pm 6.6x10^3$ CFUs) and treated with CsA. Data pooled from 3 independent experiments using n=3 larvae per condition per experiment (mean ± SD, 2-way ANOVA with Sidak's multiple comparisons test).

(D) Log_{10}-transformed CFU counts of naïve and *Shigella*-trained ($1.8x10^3 \pm 1x10^3$ CFUs) larvae injected with a lethal dose of *S. flexneri* M90T (PBS – black fill, $2.1x10^4 \pm 1.1x10^4$ CFUs, *Shigella* – green fill, $1.9x10^4 \pm 8.3x10^3$ CFUs). Data pooled from 3 independent experiments using n=3 larvae per condition per experiment (mean ± SD, 1-way ANOVA with Tukey's multiple comparisons test).

(E) Gating strategy for flow cytometry of neutrophils from Tg(*mpx*::GFP) larvae. Neutrophils were identified by *mpx-gfp* expression, followed by duplet exclusion and determination of live and dead cells (LIVE/DEAD™ Fixable Violet Stain). Live neutrophils were analysed for their total ROS production by fluorescence intensity detection of CellROX Deep Red.

(F) Normalised fluorescence of CellROX Deep Red dye neutrophils treated with DPI from naïve (black outlines) and *Shigella*-trained (green outlines, $1.4x10^3 \pm 6.7x10^2$ CFUs) larvae (Tg(*mpx*::GFP)) unstimulated (grey fills) or infected with a lethal dose of *S. flexneri* M90T (PBS – black fill, $2.6x10^4 \pm 1x10^4$ CFUs, *Shigella* – green fill, $2.6x10^4 \pm 1x10^4$ CFUs) at 6 hp2i. Data pooled from 3 experiments with n>10 larvae per condition per experiment (mean ± SD, ****p < 0.0001, 1-way ANOVA with Tukey's multiple comparisons test). a.u.: arbitrary units.

(G) Log_{10}-transformed CFU counts of naïve and *Shigella*-trained ($1.4x10^3 \pm 6.7x10^2$ CFUs) larvae injected with a lethal dose of *S. flexneri* M90T (PBS – black fill, $2.6x10^4 \pm 1x10^4$ CFUs, Shigella – green fill, $2.6x10^4 \pm 1x10^4$ CFUs) and treated with DPI. Data pooled from 3 independent experiments using n=3 larvae per condition per experiment (mean ± SD, 1-way ANOVA with Tukey's multiple comparisons test).

(H) Log_{10}-transformed CFU counts of naïve and β-glucan-trained larvae injected with a lethal dose of *S. flexneri* M90T (PBS – $2x10^4 \pm 1.3x10^4$ CFUs, β-glucan – $2.7x10^4 \pm 9.8x10^3$ CFUs). Data pooled from 3 independent experiments using n=3 larvae per condition per experiment (mean ± SD, 1-way ANOVA with Tukey's multiple comparisons test).

(I) Normalised fluorescence of GFP driven from *mpx* expression in neutrophils from naïve and *Shigella*-trained larvae (Tg(*mpx*::GFP)) infected with a lethal dose of *S. flexneri* M90T at 6 hp2i. Data pooled from 5 experiments with n>10 larvae per condition per experiment (mean ± SD, ****p < 0.0001, 1-way ANOVA with Tukey's multiple comparisons test). a.u.: arbitrary units.

Data file S1, related to Figure 5 – Gene annotation from ChIP-seq analysis of PBS treated larvae.

Data file S2, related to Figure 5 – Gene annotation from ChIP-seq analysis of *Shigella* ΔT3SS-trained neutrophils.

Data file S3, related to Figure 5 – Gene annotation from ChIP-seq analysis of *Shigella*-trained neutrophils.
